# ART in Europe, 2019: results generated from European registries by ESHRE^†^

**DOI:** 10.1093/humrep/dead197

**Published:** 2023-10-17

**Authors:** The European IVF Monitoring Consortium (EIM) for the European Society of Human Reproduction and Embryology (ESHRE), Orion Gliozheni, Eduard Hambartsoumian, Heinz Strohmer, Elena Petrovskaya, Oleg Tishkevich, Diane De Neubourg, Kris Bogaerts, Devleta Balic, Irena Antonova, Evelina Cvetkova, Karel Rezabek, John Kirk, Deniss Sõritsa, Mika Gissler, Sari Pelkonen, Imene Mansouri, Jacques de Mouzon, Andreas Tandler-Schneider, Markus Kimmel, Nikos Vrachnis, Janos Urbancsek, G Kosztolanyi, Hilmar Bjorgvinsson, Mary Wingfield, Joyce Leyden, Giulia Scaravelli, Roberto de Luca, Vyacheslav Lokshin, Sholpan Karibayeva, Valerija Agloniete, Raminta Bausyte, Ieva Masliukaite, Caroline Schilling, Jean Calleja-Agius, Veaceslav Moshin, Tatjana Motrenko Simic, Dragana Vukicevic, Jesper M J Smeenk, Zoranco Petanovski, Liv Bente Romundstad, Anna Janicka, Carlos Calhaz-Jorge, Joana Maria Mesquita Guimaraes, Patricia Duarte e Silva, Vladislav Korsak, Snezana Vidakovic, Ladislav Marsik, Borut Kovacic, Irene Cuevas Saiz, Fernando Prados Mondéjar, Christina Bergh, Sandra Toitot, Mischa Schneider, Mete Isikoglu, Basak Balaban, Mykola Gryshchenko, Elliot Bridges, Amanda Ewans, Jesper Smeenk, Christine Wyns, Christian De Geyter, Markus Kupka, Christina Bergh, Irene Cuevas Saiz, Diane De Neubourg, Karel Rezabek, Andreas Tandler-Schneider, Ionna Rugescu, Veerle Goossens

**Affiliations:** Elisabeth Twee Steden Ziekenhuis, Tilburg, The Netherlands; Cliniques universitaires Saint-Luc, Université Catholique de Louvain, Brussels, Belgium; Reproductive Medicine and Gynecological Endocrinology (RME), University Hospital, University of Basel, Basel, Switzerland; Department of Obstetrics and Gynecology, University Hospital, LMU Munich, Munich, Germany; Department of Obstetrics and Gynecology, Institute of Clinical Sciences, Göteborg University, Göteborg, Sweden; Hospital General Universitario de Valencia, Valencia, Spain; Center for Reproductive Medicine, University of Antwerp—Antwerp University Hospital, Edegem, Belgium; Department of Gynaecology, Obstetrics and Neonatology First Faculty of Medicine, Charles University and General University Hospital, Prague, Czech Republic; Fertility Center Berlin, Berlin, Germany; National Transplant Agency, Bukarest, Romania; ESHRE Central Office, Grimbergen, Belgium

**Keywords:** IVF, ICSI, IUI, egg donation, frozen embryo transfer, surveillance, vigilance, registry, data collection, fertility preservation

## Abstract

**STUDY QUESTION:**

What are the data and trends on ART and IUI cycle numbers and their outcomes, and on fertility preservation (FP) interventions, reported in 2019 as compared to previous years?

**SUMMARY ANSWER:**

The 23rd ESHRE report highlights the rising ART treatment cycles and children born, alongside a decline in twin deliveries owing to decreasing multiple embryo transfers; fresh IVF or ICSI cycles exhibited higher delivery rates, whereas frozen embryo transfers (FET) showed higher pregnancy rates (PRs), and reported IUI cycles decreased while maintaining stable outcomes.

**WHAT IS KNOWN ALREADY:**

ART aggregated data generated by national registries, clinics, or professional societies have been gathered and analyzed by the European IVF-Monitoring (EIM) Consortium since 1997 and reported in a total of 22 manuscripts published in *Human Reproduction* and *Human Reproduction Open.*

**STUDY DESIGN, SIZE, DURATION:**

Data on medically assisted reproduction (MAR) from European countries are collected by EIM for ESHRE each year. The data on treatment cycles performed between 1 January and 31 December 2019 were provided by either national registries or registries based on initiatives of medical associations and scientific organizations or committed persons in one of the 44 countries that are members of the EIM Consortium.

**PARTICIPANTS/MATERIALS, SETTING, METHODS:**

Overall, 1487 clinics offering ART services in 40 countries reported, for the second time, a total of more than 1 million (1 077 813) treatment cycles, including 160 782 with IVF, 427 980 with ICSI, 335 744 with FET, 64 089 with preimplantation genetic testing (PGT), 82 373 with egg donation (ED), 546 with IVM of oocytes, and 6299 cycles with frozen oocyte replacement (FOR). A total of 1169 institutions reported data on IUI cycles using either husband/partner’s semen (IUI-H; n = 147 711) or donor semen (IUI-D; n = 51 651) in 33 and 24 countries, respectively. Eighteen countries reported 24 139 interventions in pre- and post-pubertal patients for FP, including oocyte, ovarian tissue, semen, and testicular tissue banking.

**MAIN RESULTS AND THE ROLE OF CHANCE:**

In 21 countries (21 in 2018) in which all ART clinics reported to the registry 476 760 treatment cycles were registered for a total population of approximately 300 million inhabitants, allowing the best estimate of a mean of 1581 cycles performed per million inhabitants (range: 437–3621). Among the reporting countries, for IVF the clinical PRs per aspiration slightly decreased while they remained similar per transfer compared to 2018 (21.8% and 34.6% versus 25.5% and 34.1%, respectively). In ICSI, the corresponding PRs showed similar trends compared to 2018 (20.2% and 33.5%, versus 22.5% and 32.1%) When freeze-all cycles were not considered for the calculations, the clinical PRs per aspiration were 28.5% (28.8% in 2018) and 26.2% (27.3% in 2018) for IVF and ICSI, respectively. After FET with embryos originating from own eggs, the PR per thawing was at 35.1% (versus 33.4% in 2018), and with embryos originating from donated eggs at 43.0% (41.8% in 2018). After ED, the PR per fresh embryo transfer was 50.5% (49.6% in 2018) and per FOR 44.8% (44.9% in 2018). In IVF and ICSI together, the trend toward the transfer of fewer embryos continues with the transfer of 1, 2, 3, and ≥4 embryos in 55.4%, 39.9%, 2.6%, and 0.2% of all treatments, respectively (corresponding to 50.7%, 45.1%, 3.9%, and 0.3% in 2018). This resulted in a reduced proportion of twin delivery rates (DRs) of 11.9% (12.4% in 2018) and a similar triplet DR of 0.3%. Treatments with FET in 2019 resulted in twin and triplet DR of 8.9% and 0.1%, respectively (versus 9.4% and 0.1% in 2018). After IUI, the DRs remained similar at 8.7% after IUI-H (8.8% in 2018) and at 12.1% after IUI-D (12.6% in 2018). Twin and triplet DRs after IUI-H were 8.7% and 0.4% (in 2018: 8.4% and 0.3%) and 6.2% and 0.2% after IUI-D (in 2018: 6.4% and 0.2%), respectively. Eighteen countries (16 in 2018) provided data on FP in a total number of 24 139 interventions (20 994 in 2018). Cryopreservation of ejaculated sperm (n = 11 592 versus n = 10 503 in 2018) and cryopreservation of oocytes (n = 10 784 versus n = 9123 in 2018) were most frequently reported.

**LIMITATIONS, REASONS FOR CAUTION:**

Caution with the interpretation of results should remain as data collection systems and completeness of reporting vary among European countries. Some countries were unable to deliver data about the number of initiated cycles and/or deliveries.

**WIDER IMPLICATIONS OF THE FINDINGS:**

The 23rd ESHRE data collection on ART, IUI, and FP interventions shows a continuous increase of reported treatment numbers and MAR-derived livebirths in Europe. Although it is the largest data collection on MAR in Europe, further efforts toward optimization of both the collection and the reporting, from the perspective of improving surveillance and vigilance in the field of reproductive medicine, are awaited.

**STUDY FUNDING/COMPETING INTEREST(S):**

The study has received no external funding and all costs are covered by ESHRE. There are no competing interests.

## Introduction

This is the 23rd annual report of the European IVF-Monitoring (EIM) Consortium under the umbrella of ESHRE, assembling the data on ART, IUI, and fertility preservation (FP) reported by 40 participating European countries in 2019 (Supplementary Table S1 and Supplementary Data File S1).

Eighteen previous annual reports published in *Human Reproduction* (https://www.eshre.eu/Data-collection-and-research/Consortia/EIM/Publications.aspx) and four in *Human Reproduction Open* (De Geyter *et al.*, 2020a; Wyns *et al.*, 2020, 2021, 2022), covered data on treatment cycles collected yearly from 1997 to 2018. As in previous reports, the manuscript contains the five most relevant tables. Twenty additional supplementary tables (Supplementary Tables S1, S2, S3, S4, S5, S6, S7, S8, S9, S10, S11, S12, S13, S14, S15, S16, S17, S18, S19, and S20) are available online on the publisher’s homepage. To allow easy comparison and assessment of trends, the presentation of the data is consistent with previous reports. For the fourth consecutive year, data on FP were collected and added to this report.

## Materials and methods

Data were collected on an aggregate basis and were provided by 40 European countries, covering treatments with IVF, ICSI, frozen embryo transfer (FET), egg donation (ED), IVM, preimplantation genetic testing (PGT; pooled data), frozen oocyte replacement (FOR), IUI with husband’s/partner’s semen (IUI-H), and with donor semen (IUI-D). The report includes treatments that started between 1 January and 31 December in 2019. Data on pregnancies and deliveries represent the outcomes of treatments performed in 2019. Aggregated data on FP include numbers and types of cryopreserved material and interventions for the use of cryo-stored material between 1 January and 31 December in 2019.

The national representatives of the 44 countries being members of the EIM consortium were asked to fill out the survey with the same data requirements as in 2018. A total of 10 modules on specific topics/questions were sent using software designed for the requirements of this data collection (Evidenze, former: Dynamic Solutions, Barcelona, Spain). Any identified inconsistency was clarified through direct contact between the administrator of the ESHRE central office (VG) and the national representative.

The data were analyzed and presented similarly to previous reports. Footnotes to the tables were added to clarify some results reported by individual countries, when applicable.

The terminology used was based on the glossary of The International Committee for Monitoring Assisted Reproductive Technology (Zegers-Hochschild *et al.*, 2017).

## Results

### Participation and data completeness

Table 1 shows the number of clinics providing ART services with the different treatment modalities they offer and institutions performing IUI (IUI-H and IUI-D). Compared to 2018, the total number of reporting clinics (1488 versus 1422 in 2018) and the number of reported treatments (1 077 813 versus 1 007 598 in 2018, +7.0%) increased. Among the 51 European countries, 44 are EIM members including 28 that were members of the European Union (EU) at that time and 40 (39 in 2018) provided data (Supplementary Table S1). Non-EIM members are mainly small countries not offering ART services. Croatia, Cyprus, Georgia, and Romania did not deliver data in 2019 (9.1% of EIM members). In 21 countries (52.5% of reporting countries), all ART clinics participated in the reporting. Among 1774 (1552 in 2018) known IVF clinics in Europe, 1488 clinics reported data sets (83.9% versus 91.6% in 2018). The main differences with 2018 can be explained by the renewed, but still limited, participation of Turkey in 2019. As in 2018, the four European countries with the largest treatment numbers in 2019 were Russia (161 166; 155 949 in 2018), Spain (137 276; 140 498 in 2018), France (118 394; 106 884 in 2018), and Germany (107 136; 105 328 in 2018).

**Table 1.** Treatment frequencies after ART in European countries in 2019.

	ART clinics in the country												Cycles/million	
Country	IVF Clinics	Included IVF clinics	IUI labs	Included IUI labs	IVF	ICSI	FET	PGT	ED	IVM	FOR	All	Women 15-45	Population
Albania	10	1	10		0	94	73	16	10	0	1	194		
Armenia	6	5	6	4	654	727	2143	33	544	0	0	4101		
Austria	30	30	0	0	1839	5485	3485	0	0	0	0	10 809	6690	1225
Belarus	8	7	10	7	1188	2279	1316	89	140	0	7	5019		
Belgium	18	18	29	29	2578	13 812	14 597	1476	1700	184	93	34 440	16 093	2960
Bosnia-Herzegovina, Federation part	9	1			0	91	71	0	0	0	0	162		
Bulgaria	36	36	37	37	798	5684	1832	347	763	0	0	9424	7327	1344
Czech Republic	46	46			652	14 168	16 174	1849	5874	0	0	38 717	19 393	3621
Denmark	19	19	51	50	7657	6350		663	1145	0	44	15 859	14 762	2715
Estonia	6	6	6	6	669	1458	1142	54	246	0	6	3575	16 555	2892
Finland	16	16	20	20	2374	1619	3575	172	692	0	0	8432	8564	1518
France	103	103	176	176	23 733	45 080	45 312	1968	1596	123	582	118 394	10 007	1751
Germany	139	133			21 949	54 348	30 430		0	0	409	107 136		
Greece	41	40	41	40	2370	13 282	5607	475	4828	16	116	26 694		
Hungary	14	11	21	13	0	8555	1616		0	0	0	10 171		
Iceland	1	1	1	1	256	211	377	0	106	0	0	950	13 801	2736
Ireland	9	2	10	3	607	722	814	61	9	0	1	2214		
Italy	189	189	299	299	7387	42 937	21 796	4709	6848	0	1361	85 038	7976	1384
Kazakhstan	23	18	23	18	2750	5598	4860	769	1771	0	0	15 748		
Latvia	6	3	6	3	122	671	661	19	196	0	2	1671		
Lithuania	7	7	7	7	694	819	387	13	6	0	1	1920	3943	694
Luxembourg	1	1			253	499	505		0	0	0	1257	10 033	2036
Malta	4	2	5		0	161	23	0	0	0	14	198	1976	437
Moldova	5	4	5	4	0	628	415		0	0	0	1043		
Montenegro	5	5	0	5	0	611	75		0	0	0	686		
North Macedonia	2	2	8	8	200	2166	408	0	212	0	0	2986	5675	1407
Norway	11	11	11	11	3704	3418	4516	0	0	0	0	11 638	11 283	2147
Poland	44	42		42	173	16 268	13 346	1191	1476	11	694	33 159		
Portugal	25	25	27	27	2425	4028	3125	644	1815	21	140	12 198	6315	1183
Russia	299	219			35 645	52 348	50 864	11 158	10 156	98	897	161 166		
Serbia	18	6	18	6	1000	1009	465		0	10	7	2491		
Slovakia	12	12	5	5	0	5317			236	0	0	5553	5137	1020
Slovenia	3	3	2	2	1313	1956	1721	68	1	0	2	5061	14 184	2407
Spain	244	242	304	304	5807	42 316	30 357	22 190	35 674	37	895	137 276		
Sweden	19	18			6190	5938	7625	538	331	0	0	20 622		
Switzerland	28	28			765	5276	5124	620	0	0	0	11 785	7478	1413
The Netherlands	15	15			6240	7101	14 257	615	0	0	0	28 213	9081	1639
Turkey	167	26	167	25	170	19 702	8433	4778	0	0	15	33 098		
Ukraine	50	49	17	17	514	14 515	13 849	7397	2113	0	14	38 402		
UK	86	86	100		18 106	20 733	24 368	2177	3885	46	998	70 313	5595	1056
All	1774	1488	1422	1169	160 782	427 980	335 744	64 089	82 373	546	6299	1 077 813	8706	1581

Treatment cycles in IVF and ICSI refer to initiated cycles. For Belgium treatment cycles refer to aspirations, not taking into account 863 aspirations where the treatment performed is not known. For Bosnia-Herzegovina (Federation) and Serbia, treatment cycles refer to aspirations. For Belgium and Serbia, the total number of initiated cycles was only available for IVF and ICSI together, being 19 224 and 2012, respectively. For Hungary only the total number of initiated cycles was available for IVF and ICSI together, being 8555. This number was counted as ICSI in this table. Treatment cycles in frozen embryo transfer (FET) refer to thawings. For Finland, Luxembourg, Sweden, and the Netherlands, treatment cycles refer to transfers. Treatment cycles in preimplantation genetic testing (PGT) contain both fresh and frozen cycles and refer to initiated cycles in the fresh cycles and thawings in the frozen cycles. Treatment cycles in egg donation (ED) refer to donation cycles and contain fresh and frozen cycles. ED fresh: for France and Iceland, treatment cycles refer to aspirations. ED frozen: for France, Iceland, Kazakhstan, Spain, Sweden, and the UK treatment cycles refer to aspirations. Treatment cycles in IVM refer to aspirations. Treatment cycles in frozen oocyte replacement (FOR) refer to thawings, for Finland it refers to transfers. Women of reproductive age and population were found at the following link: http://www.census.gov/population/international/data/idb/region.php.

### Size of the clinics and reporting methods

The size of reporting clinics, as calculated based on the number of fresh and frozen cycles per year, was highly variable among and within countries, as seen in previous years (Supplementary Table S2). In 2019, as in 2018, clinics with cycle numbers between 200 and 499, and 500 and 999, were the most common (25.7% and 27.8%, respectively, versus 27.3% and 26.3%). The proportion of clinics performing more than 1000 treatment cycles per year was slightly higher than in 2018 (22.3% versus 21.0% in 2018). Small clinics with fewer than 100 treatment cycles per year were present in 24 countries (21 countries in 2018).

Requirements of registries and reporting methods of the countries are shown in Supplementary Table S3. Data collection was either voluntary (15 out of 40 countries) or compulsory. Twenty-six countries reported all or a part of the treatment cycles to the national health authority. Among 19 countries with only partial reporting, data were mainly provided voluntarily (14 countries) to medical organizations (10 countries), to the national health authority (8 countries), or to a single individual who took the initiative to organize the data collection (2 countries).

In contrast, complete reporting was most often achieved when data collection was compulsory (20/21 countries) and with data communication to the national health authority (all but three countries). Transfer of the data was mainly done on an aggregate basis (24 out of 40 countries).

### Number of treatment cycles per technique and availability

In 2019, 1 077 813 treatment cycles were reported to EIM (70 215 more than in 2018, +7.0%). Since 1997, the increasing numbers of clinics reporting to EIM resulted in a total of 12 804 411 treatment cycles and the birth of more than 2 479 254 infants (Table 2). As seen in Table 1, most countries reported similar numbers of treatment cycles as in 2018. Furthermore, the largest increments in reported treatment numbers were observed in France (+11 510, +10.8%) and Ukraine (+10 081, +35.6%). The largest reduction in reported treatment cycle numbers was seen in Denmark (−4757, −23.1%).

**Table 2.** Number of institutions offering ART services, treatment cycles, and infants born after ART in Europe, 1997–2019.

Year	No. of countries	No. of centers	No. of cycles	Cycle increase (%)	No. of infants born
1997	18	482	203 225		35 314
1998	18	521	232 225	+14.3	21 433
1999	21	537	249 624	+7.5	26 212
2000	22	569	275 187	+10.2	17 887
2001	23	579	289 690	+5.3	24 963
2002	25	631	324 238	+11.9	24 283
2003	28	725	365 103	+12.6	68 931
2004	29	785	367 056	+0.5	67 973
2005	30	923	419 037	+14.2	72 184
2006	32	998	458 759	+9.5	87 705
2007	33	1029	493 420	+7.7	96 690
2008	36	1051	532 260	+7.9	107 383
2009	34	1005	537 463	+1.0	109 239
2010	31	991	550 296	+2.4	120 676
2011	33	1314	609 973	+11.3	134 106
2012	34	1354	640 144	+4.9	143 844
2013	38	1169	686 271	+7.2	149 466
2014	39	1279	776 556	+13.1	170 163
2015	38	1343	849 811	+10.2	187 542
2016	40	1347	918 159	+8.0	195 766
2017	39	1382	940 503	+2.4	198 215
2018	39	1422	1 007 598	+7.1	215 614
2019	40	1488	1 077 813	+7.0	203 665
Total			12 804 411		2 479 254

Table 1 shows the number of treatment cycles per technique in 2019: ICSI remains the most used technology (427 980, 39.7%), versus 400 375 (39.7%) in 2018. Cycles with IVF, FET, ED, FOR, PGT, and IVM represented 14.9%, 31.2%, 7.6%, 0.6%, 5.9%, and 0.0005% of all cycles, respectively, in 2019. The distribution of the available techniques remained similar to 2018 (respectively, 16.2%, 30.7%, 8%, 0.5%, 4.8%, and 0.0005%). Reported cycle numbers with ICSI, FET, ED, PGT, IVM, and FOR increased, and only those with IVF decreased (−1.3%).

The steepest rise in treatment numbers was observed for PGT (+32.7%; +29.5% in 2018), FOR (+15.7%; +4.5% in 2018), and FET (+8.5%; +14.0% in 2018).

**Figure 1.**
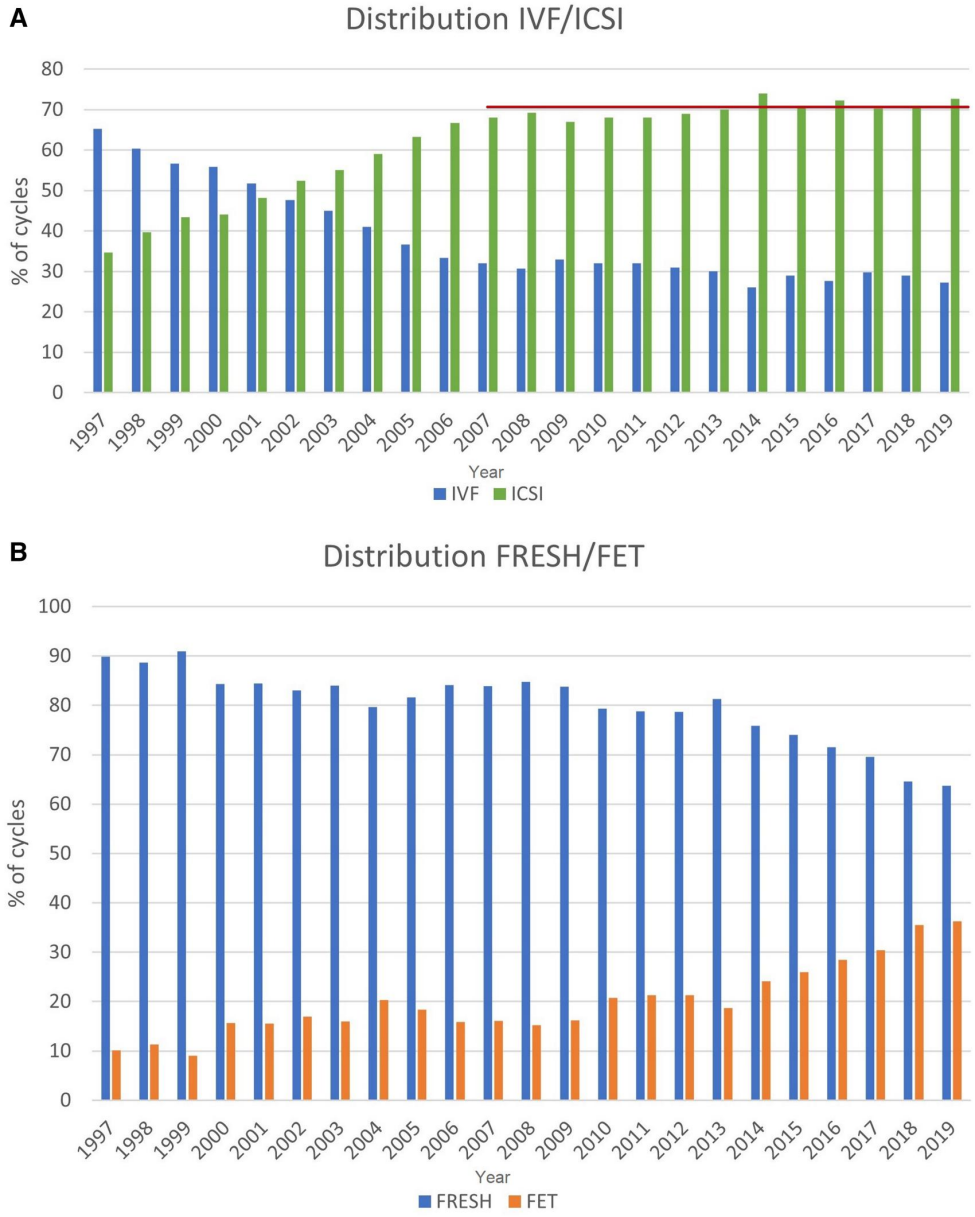
**Distribution of treatments in Europe, 1997–2019.** (**A**) Proportion of IVF versus ICSI cycles. (**B**) Proportion of fresh versus frozen cycles. FET: frozen embryo transfer.

The highest proportions of FET treatments (calculated as FET/(FET + ICSI + IVF)) were reached in Armenia (60.8%), Czech Republic (52.2%), The Netherlands (51.7%), Ukraine (48.0%), Finland (47.2%), Belgium (47.1%), and Switzerland (45.9%) with an overall proportion of 36.3% and comparable to 35.5% in 2018 (Fig. 1A and B).

Figure 1A shows the evolution and continuing preponderance of ICSI over conventional IVF. Among a total of 588 762 fresh treatments (ICSI + IVF), 72.7% (71.1% in 2018) were done with ICSI.

The number of treatment cycles per million women of reproductive age and per million inhabitants is shown in Table 1 and Supplementary Table S4. Availability of ART treatments was calculated for the 21 countries with full coverage (Supplementary Table S4) showing a huge variability in availability when all techniques are considered (range per million women aged 15–45 years: 3943 in Lithuania to 19 393 in Czech Republic). Corresponding proportions of newborns resulting from ART ranged from 1.2% to 6.3% of all newborns in these countries. Among the countries with complete reporting to the national registry, proportions of ART infants above 5% were reached in Belgium, the Czech Republic, Denmark, Estonia, and Iceland.

### Pregnancies and deliveries after treatment

Table 3 shows pregnancy and delivery rates after IVF or ICSI and after FET (after both IVF and ICSI). Outcome data were calculated per aspiration, rather than per initiated cycle, as the numbers of initiated cycles have often been reported incompletely.

**Table 3.** Results after ART in 2019.

	IVF				ICSI			FET				
Country	Initiated Cycles IVF + ICSI	Aspirations	Pregnancies per aspiration (%)	Deliveries per aspiration (%)	Aspirations	Pregnancies per aspiration (%)	Deliveries per aspiration (%)	Thawings FET	Pregnancies per thawing (%)	Deliveries per thawing (%)	ART infants^†^	ART infants per national births (%)
Albania	94	0			94	33.0	27.7	73	27.4	20.5	63	
Armenia	1381	621	21.3	19.0	693	16.0	13.4	2143	38.0	32.8	1232	2.9
Austria	7324							3485	33.1		2601	
Belarus	3467	1014	31.7	18.5	1948	26.8	13.4	1316	37.7	24.3	930	
Belgium	19 224	2578	21.1	15.6	13 812	21.8	15.9	14 597	28.6	19.9	6271	5.3
Bosnia-Herzegovina, Federation part	91	0			91	40.7	28.6	71	22.5	15.5	40	
Bulgaria	6482	798	20.4	17.4	5684	17.3	15.4	1832	40.2	33.9		
Czech Republic	14 820	357	27.5	23.2	13 886	21.3	14.0	16 174	31.0	18.9	7037	6.2
Denmark	14 007	6823	20.2	12.3	5516	22.0	15.5				3871	6.3
Estonia	2127	657	19.8	16.7	1424	25.4	19.6	1142	30.2	22.3	801	5.7
Finland	3993	2228	25.1	19.8	1526	19.8	15.6				1735	
France	68 813	20 952	18.4	15.7	41 871	19.1	16.4	45 312	25.4	21.2	22 007	2.9
Germany	76 297	19 610	26.6	19.7	49 330	25.1	18.4	30 430	28.6	20.3	22 405	
Greece	15 652	2298	20.5	15.4	13 118	17.4	10.6	5607	44.9	28.3	6290	7.5
Hungary	8555							1616	24.3	18.3	1944	2.2
Iceland	467	234	24.4	16.2	202	26.2	21.8	377	42.4	33.2	243	5.5
Ireland	1329	553	34.2	25.1	653	35.2	28.3	814	42.5	32.2	1166	1.9
Italy	50 324	6730	19.9	13.9	39 360	16.3	10.7	21 796	31.0	20.2	13 020	3.1
Kazakhstan	8348	2750	22.2	16.2	5598	21.1	17.3	4860	46.0	35.0	4484	
Latvia	793	122	23.8	16.4	664	23.6	15.4	661	44.9	30.6	401	
Lithuania	1513	685	53.1	24.1	789	46.1	11.5	387	48.3	7.2	332	1.2
Luxembourg	752	230	20.9	16.1	474	22.6	16.5	0			255	3.5
Malta	161							23	39.1	30.4	12	
Moldova	628				610			415	33.7	24.8		
Montenegro	611				589	31.4	25.1	75	41.3	38.7	218	3.0
North Macedonia	2366	160	45.0	23.8	2076	34.6	21.7	408	46.1	19.9	701	2.4
Norway	7122	3540	27.5	23.9	3231	25.0	22.3	4516	28.0	23.2		
Poland	15 608	173	26.0	20.8	14 503	22.3	13.9	12 875	37.5	25.6	6177	
Portugal	6453	2303	23.6	17.3	3674	19.9	14.8	3125	37.0	25.6	2710	3.1
Russia	87 993	34 842	26.9	18.1	51 078	23.2	14.6	50 864	42.2	27.0	36 172	2.4
Serbia	2012	1000	27.0	19.5	1009	26.3	18.3	465	24.1	15.3	579	0.9
Slovakia	5317											
Slovenia	3269	1288	31.2	24.3	1860	24.9	20.1	1721	35.6	26.7	1234	
Spain	48 123	5344	22.1	16.4	37 976	18.2	13.5	30 357	37.5	26.5	31 982	8.9
Sweden	12 128	5845	26.6	22.1	5555	25.5	21.5				5448	4.7
Switzerland	6041	719	21.1	16.1	4861	18.8	14.2	5124	34.4	24.8	2212	2.6
The Netherlands	13 341	5384	30.5	22.3	6483	33.7	25.1					
Turkey	19 872	136	44.9	29.4	11 075	26.9	21.2	8433	49.5	39.1	7582	0.6
Ukraine	15 029	488	33.4	23.0	14 133	17.8	13.7	13 849	50.1	41.4	11 823	4.0
UK	38 839	16 072			20 474			24 368				
All	590 766	146 534	21.8	16.0	375 920	20.2	14.5	309 311	32.3	22.7	203 978	3.0

Total rates refer to these countries where all data were reported for the given technique. For IVF and ICSI, there were for Austria, Belarus, Belgium, the Czech Republic, Finland, France, Germany, Greece, Ireland, Kazakhstan, Latvia, Lithuania, Malta, Portugal, Russia, Slovenia, Spain, and Turkey, respectively, 1640, 219, 13, 2, 682, 10, 20, 36, 9, 115, 4, 84, 5, 6, 376, 2, 168, and 2 deliveries with unknown outcome. These were accepted as singletons to calculate the ART infants. For frozen embryo transfer (FET), there were for Austria, Belarus, Belgium, the Czech Republic, Finland, France, Germany, Greece, Kazakhstan, Latvia, Lithuania, Malta, Portugal, Russia, and Turkey, respectively, 961, 80, 19, 7, 892, 10, 40, 38, 67, 40, 4, 7, 4, 835, and 2 deliveries with unknown outcome. These were accepted as singletons to calculate the ART infants. For ED, there were for Belarus, Belgium, the Czech Republic, Finland, France, Greece, Kazakhstan, Latvia, Portugal, and Russia, respectively, 5, 1, 6, 163, 1, 72, 38, 9, 1, and 104 deliveries with unknown outcome. These were accepted as singletons to calculate the ART infants. For PGT, there were for France, Greece, and Russia, respectively, 2, 1, and 44 deliveries with unknown outcome. These were accepted as singletons to calculate the ART infants.

† ART infants also include preimplantation genetic testing (PGT) and egg donation.

Among the 40 reporting countries, 31 were able to provide both pregnancy and delivery rates per aspiration after IVF. After ICSI, 34 countries were able to provide both pregnancy and delivery rates per aspiration. For FET when considering thawing cycles, 33 countries were able to report pregnancy and delivery rates (32 in 2018). Supplementary Table S4 shows the number of deliveries for the 21 countries with full coverage.

Significant variation in pregnancy and delivery rates (for all types of treatment cycles) was observed among different countries, as in previous years.

**Figure 2.**
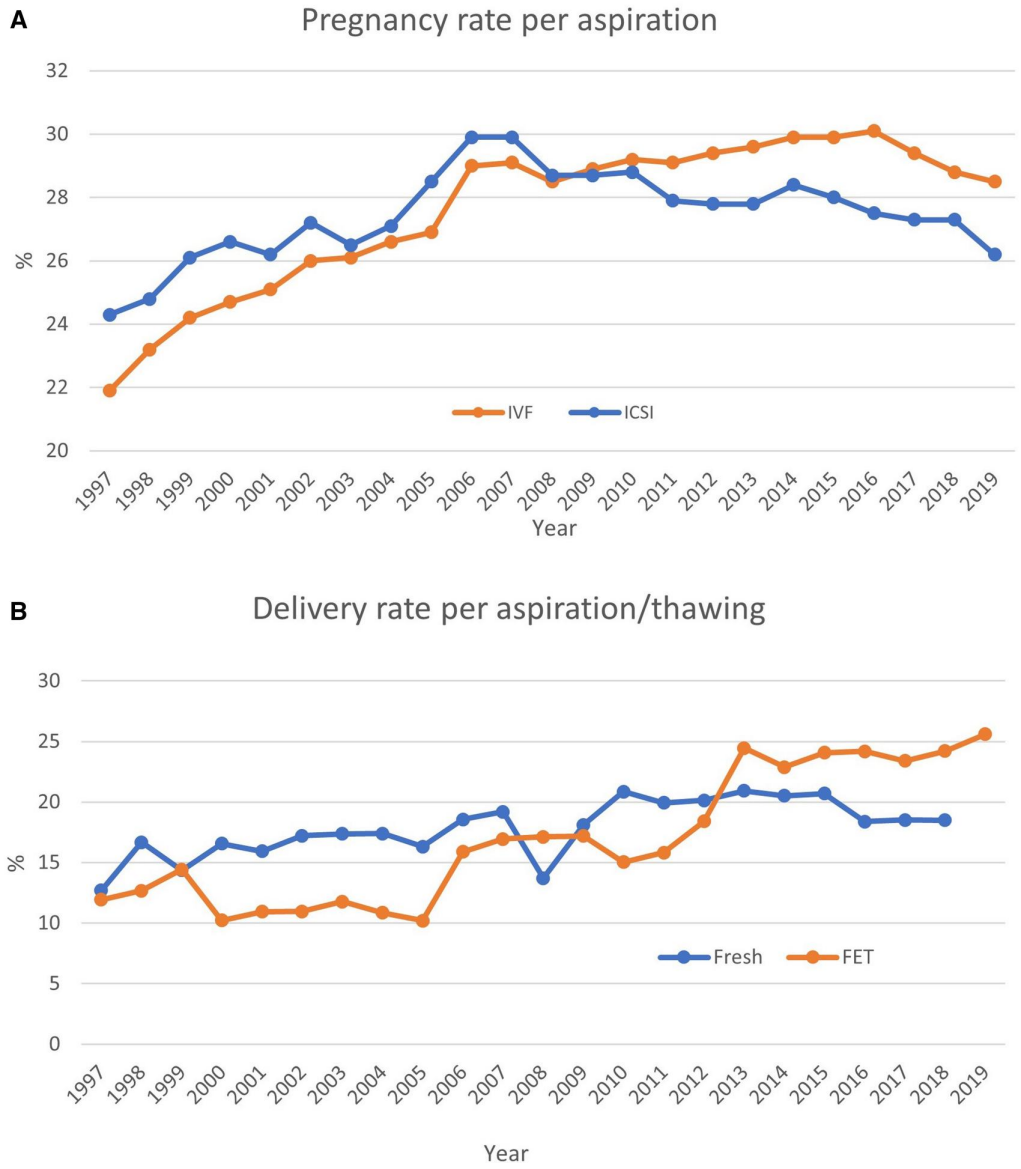
**Pregnancy and delivery rates per aspiration in Europe, 1997–2019.** (**A**) Pregnancy rates for IVF versus ICSI cycles. (**B**) Delivery rates for fresh versus frozen cycles. FET: frozen embryo transfer.

**Figure 3.**
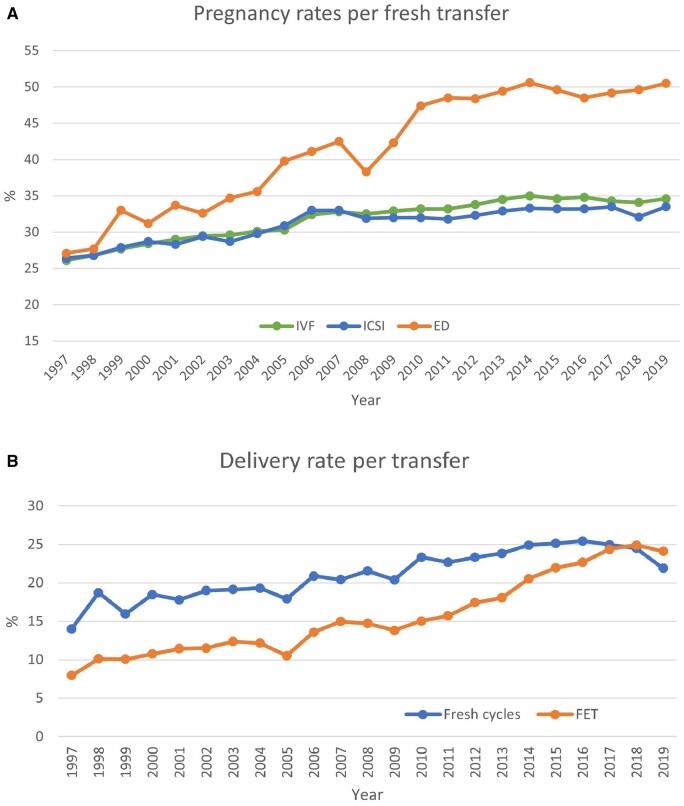
**Pregnancy and delivery rates per transfer in Europe, 1997–2019.** (**A**) Pregnancy rates for IVF versus ICSI and ED cycles. (**B**) Delivery rates for fresh versus frozen cycles. ED, egg donation.

Per aspiration, pregnancy rates (PRs) are shown in Fig. 2A and ranged from 16.0% to 53.1%. The delivery rates are shown in Fig. 2B and ranged from 10.6% to 29.4% in fresh cycles after IVF or ICSI (including the freeze-all cycles whether performed or not by the countries) (Table 3). For FET, pregnancy and delivery rates per thawing varied between 22.5% and 50.1% and between 7.2% and 41.4%, respectively. Overall, while higher pregnancy and delivery rates were recorded for FET cycles (per thawing) than for both fresh IVF and ICSI cycles (per aspiration) (Table 3; Supplementary Table S7), PRs per transfer in fresh cycles remained at the same level (34.6% for IVF and 33.5% for ICSI; Fig. 3A), but were slightly higher in FET cycles (35.8%), as were delivery rates per transfer (25.3% for IVF, 24.1% for ICSI, and 25.6% for FET), as in 2018 (Supplementary Tables S5, S6, and S7 and Fig. 3B).

When considering the developmental stage of replaced embryos, the data showed PRs for blastocyst transfers to be higher (39.4%) than for cleavage-stage embryos (26.5%) in fresh IVF and ICSI cycles together (it was not possible to distinguish between IVF and ICSI). A similar picture was seen in FET cycles: 40.5% PRs for blastocyst transfers versus 26.9% for cleavage-stage embryos.

Cycle numbers, aspirations, transfers, pregnancies, and deliveries in IVF, ICSI, and FET (after both IVF and ICSI) by country are presented in Supplementary Tables S5, S6, and S7.

For the sixth year, ‘freeze-all’ cycles were collected (Supplementary Tables S5 and S6) including either freezing of all oocytes reported by 11 countries for IVF (11 in 2018 and 10 in 2017) and 19 countries for ICSI (17 in 2018 and 17 in 2017), or of all embryos by 26 countries for IVF (23 in 2018 and 22 in 2017) and 27 countries for ICSI (25 in 2018 and 27 in 2017). The highest proportions of freeze-all cycles per aspiration (oocytes and embryos together) were 61.0% (29.8% in 2018) and 61.9% (41.7% in 2018), respectively, for IVF and ICSI.

ED cycle numbers were available for 20 countries (23 in 2018) although 27 (27 in 2018) provided outcome data (Supplementary Table S8). The highest numbers of ED cycles were reported from Spain, the Czech Republic, and Russia, as in 2018. The number of aspirations of donated oocytes was 34 406 (36 938 in 2018) resulting in 22 932 fresh transfers (24 148 in 2018), while the number of replacements of frozen oocytes (FOR) was 16 122 (16 130 in 2018). The PR per fresh ET was 50.5% (49.6% in 2018) for freshly donated oocytes and 44.8% (44.9% in 2018) for thawed oocytes. High variability was seen between countries, ranging from 31.5% to 100% for fresh oocytes and from 23.2% to 80.0% for thawed oocytes, as in previous years, although sometimes small numbers were observed. Overall (including also the transfers of frozen embryos), 25 156 deliveries were reported with donated eggs (25 760 in 2018 and 21 312 in 2017). Compared to cycles with own oocytes, pregnancy and delivery rates per transfer were higher for fresh (IVF and ICSI) and FET cycles together.

### Age distribution

Supplementary Tables S9 and S10 showed that the age distributions of women treated with IVF and ICSI, respectively, varied between countries. Some countries were not able to provide age categories (nine for IVF and six for ICSI). The highest percentage of women aged 40 years and older undergoing aspiration for IVF was reported in Greece (as in 2018), whereas the highest percentage of women aged <34 years was reported in Ukraine (as in 2018). For ICSI, the highest percentage of women aged 40 years and older undergoing aspiration was also reported in Greece (as in 2018), whereas the highest percentage of women undergoing aspiration aged <34 years was recorded in Sweden (as in 2018). An age-dependent decrease in pregnancy and delivery rates for IVF and ICSI cycles was reported, as expected. Pregnancy and delivery rates in women aged 40 years and older ranged between 6.5% and 56.7%, and 1.5% and 23.8%, respectively. These age-related declines were also visible in FET cycles (Supplementary Table S11) with recorded pregnancy and delivery rates among women aged 40 years and older ranging from 7.7% to 42.5% and 0% to 35.5%, respectively.

As seen in Supplementary Table S12, the age of the recipient women had little influence on the outcomes of ED cycles.

### Numbers of embryos transferred and multiple births

Differences in the number of embryos replaced per transfer after IVF and ICSI together, with multiple birth rates per subgroups defined by the number of embryos replaced, are presented in Table 4.

**Table 4.** Number of embryos transferred after ART and deliveries in 2019.

	IVF + ICSI								FET		
Country	Transfers	1 embryo (%)	2 embryos (%)	3 embryos (%)	4+ embryos (%)	Deliveries	Twin (%)	Triplet (%)	Deliveries	Twin (%)	Triplet (%)
Albania	66	1.5	98.5	0.0	0.0	26	26.9	3.8	15	20.0	0.0
Armenia	544	26.1	52.4	21.5	0.0	211	16.1	3.3	702	3.6	0.6
Austria	5714	73.3	26.6	0.1	0.0	1640			961		
Belarus	2135	32.9	59.2	7.9	0.0	449	12.2	0.9	320	17.5	0.0
Belgium	12 048	72.5	24.5	2.7	0.3	2604	5.7	0.1	2902	4.2	0.0
Bosnia-Herzegovina, Federation part	71	76.1	19.7	4.2	0.0	26	3.8	0.0	11	18.2	0.0
Bulgaria											
Czech Republic	10 151	79.9	19.9	0.2	0.0	2033	5.7	0.1	3057	6.3	0.1
Denmark	8492	83.1	16.5	0.4	0.0	1695	3.5	0.1	1830	1.7	0.0
Estonia	1500	57.6	40.3	2.1	0.0	389	9.8	0.3	255	10.6	0.4
Finland	2564	95.7	4.3	0.0	0.0	680			892		
France	40 432	60.8	36.6	2.4	0.1	10 182	8.9	0.1	9613	5.4	0.1
Germany	53 737	34.4	62.6	3.0	0.0	12 936	18.1	0.4	6163	13.0	0.5
Greece	7284	23.5	61.0	13.2	2.2	1741	20.0	0.2	1584	17.3	0.3
Hungary	6883					1354	16.8	0.8	296	13.2	1.0
Iceland	299	100.0	0.0	0.0	0.0	82	0.0	0.0	125	2.4	0.0
Ireland	977	50.7	46.9	2.5	0.0	324	11.1	0.0	262	0.4	0.0
Italy	28 731	44.8	46.7	7.8	0.7	5151	12.3	0.3	4412	5.0	0.1
Kazakhstan	4193	51.9	46.5	1.6	0.0	1414	12.6	0.3	1702	12.0	0.1
Latvia	380	83.4	16.6	0.0	0.0	122	5.9	0.0	202	6.8	0.0
Lithuania	1386	56.1	29.5	14.4	0.0	256	11.0	1.7	28	29.2	0.0
Luxembourg	499	55.7	44.3	0.0	0.0	115	4.3	0.9	125	6.4	0.0
Malta	18					5			7		
Moldova											
Montenegro	481	29.7	49.1	21.2	0.0	148	23.6	0.0	29	20.7	0.0
North Macedonia	1734	35.9	62.2	1.8	0.1	488	14.8	0.0	81	11.1	0.0
Norway											
Poland	8884	66.9	32.9	0.1	0.0	2137	6.1	0.1	3400	4.1	0.1
Portugal	3692	54.0	45.6	0.4	0.0	941	9.9	0.2	801	9.0	0.1
Russia	58 120	61.1	38.5	0.3	0.0	13 758	12.5	0.2	13 737	12.2	0.2
Serbia	1511	23.5	31.8	41.8	3.0	380	28.7	1.6	71	9.9	0.0
Slovakia											
Slovenia	2578	61.8	38.1	0.1	0.0	686	7.3	0.0	459	6.3	0.0
Spain	23 132	52.8	46.2	1.0	0.0	6020	11.0	0.1	8056	8.9	0.0
Sweden	8587	88.8	11.2	0.0	0.0	2485	2.7	0.0	2668	1.8	0.0
Switzerland	3207	72.2	26.5	1.2	0.1	808	7.7	0.4	1272	4.7	0.2
The Netherlands											
Turkey	7796	58.7	41.3	0.0	0.0	2388	11.6	0.1	3294	13.4	0.2
Ukraine	5747	41.3	53.9	4.8	0.0	2042	15.8	0.2	5734	14.1	0.0
UK	30 879	68.6	29.7	1.7	0.0						
All^a^	344 452	55.4	39.9	2.6	0.2	75 716	11.9	0.3	75 066	8.9	0.1

a Total refers only to these countries where data on number of transferred embryos and on multiplicity were reported.

FET, frozen embryo transfer.

**Figure 4.**
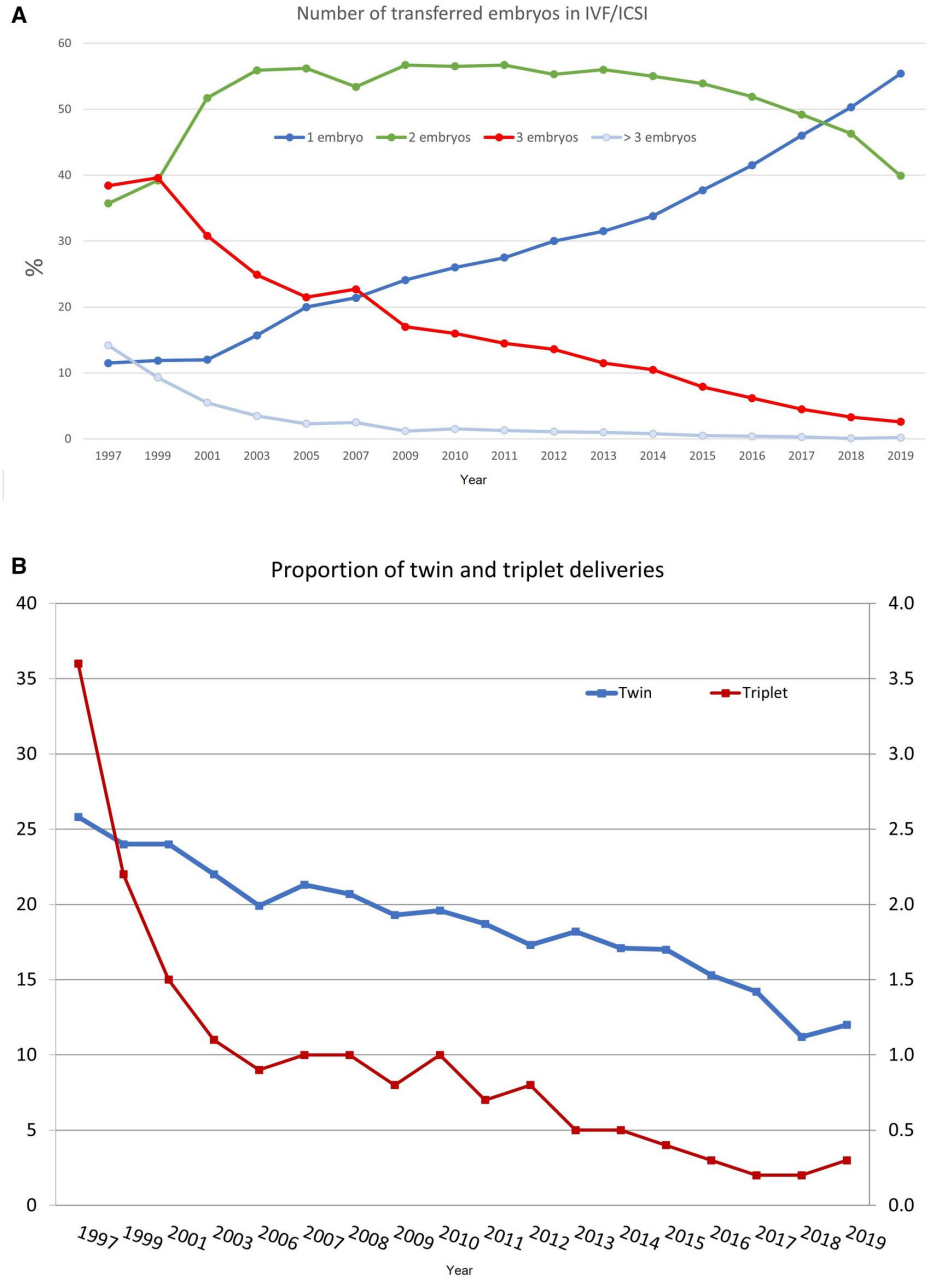
**Embryo transfer and multiple births in Europe, 1997–2019.** (**A**) Number of embryos transferred in IVF and ICSI during fresh cycles. (**B**) Percentages of twin and triplet deliveries.

Six countries reported neither the number of replaced embryos nor the multiplicity. Most transfers involved the replacement of one embryo (elective or not) (55.4% of cycles, as compared to 50.7% of single embryo replacements in 2018). The evolution of the proportions of replacements of one, two, and three or more embryos is shown in Fig. 4A.

Twenty-three countries reported more than 50% single embryo transfers (17 in 2018) with seven reporting more than 75% single embryo transfers. None of the reporting countries carried out more than 50% of their transfers with three embryos. Among the seven countries recording transfers of four or more embryos, the highest proportion was found in Serbia (3.0%; 5.5% in Greece in 2018). For the third consecutive year, the embryonic developmental stage at transfer was recorded. Taking into account that the embryo stage at transfer was unknown in 18.4% of the fresh (IVF + ICSI) cycles, 52.8% (50.1% in 2018) of the transfers were performed at the blastocyst stage. The corresponding percentage for FET was 79.1% (73.9% in 2018). Information about the embryonic developmental stage was not available with respect to the number of embryos replaced.

As a result of the decreasing number of embryos replaced per transfer, the global proportion of twin and triplet deliveries continued to decrease (Fig. 4B). Twin and triplet rates for fresh IVF and ICSI cycles together were 12.0% (range 0–26.9) and 0.3% (range: 0–3.8), respectively. Corresponding results for FET were 9.3% and 0.1%. Two countries reported rates of single embryo replacement above 95% in fresh cycles (100% in Iceland, 95.7% in Finland) and twin rates were as low as 0% (in Iceland, data from Finland were not available).

Supplementary Tables S13 and S14 provide additional information on pregnancies and deliveries. The reported incidence of pregnancy loss was 19.9% after removing the data of those countries in which pregnancy loss was not documented (19.3% in 2018) after IVF + ICSI and 21.5% (21.4% in 2018) after FET. The proportion of recorded lost to follow-up pregnancies was 8.7% (7.2% in 2018) after IVF + ICSI and 8.6% (7.2% in 2018) after FET.

### Perinatal risks and complications

**Figure 5.**
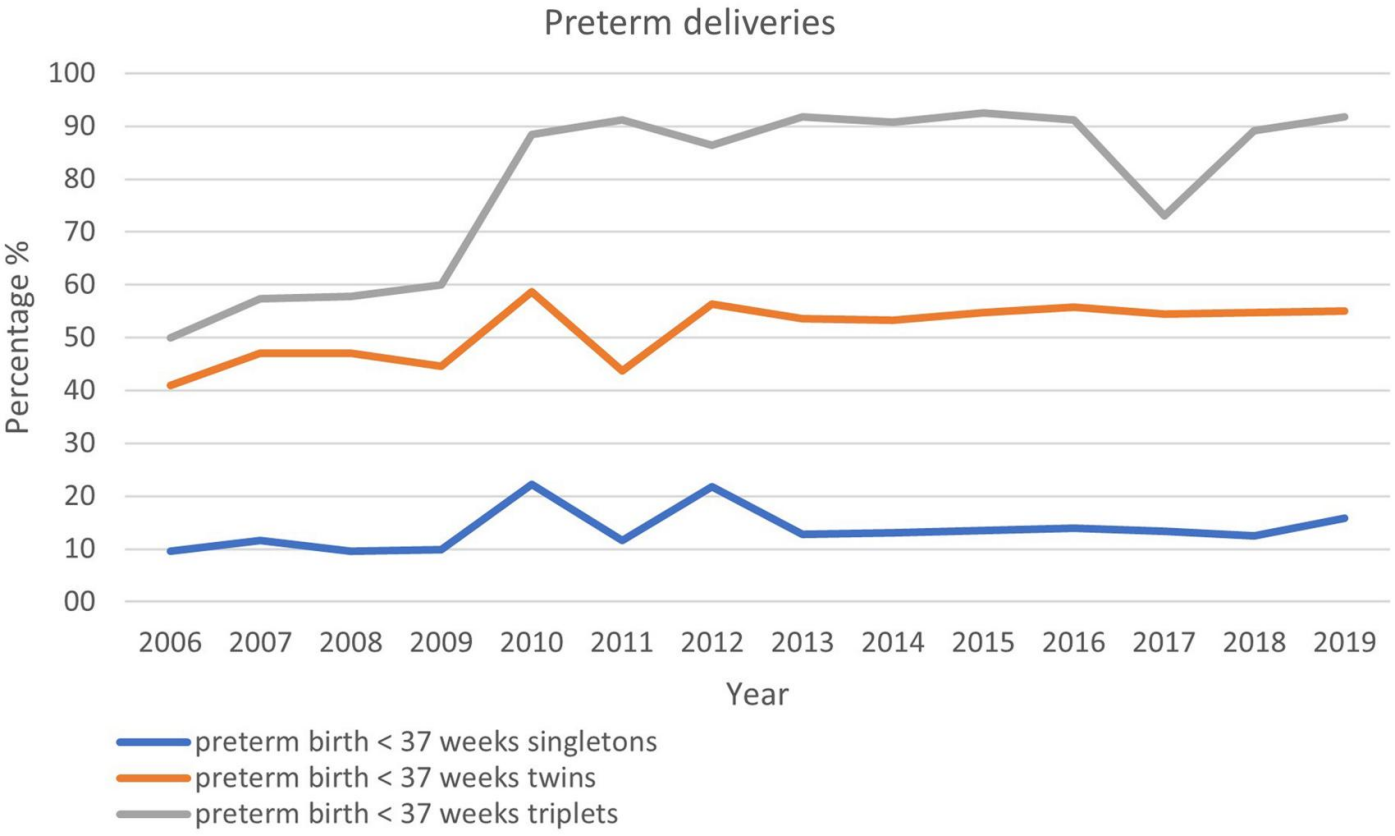
**Proportion of premature deliveries (<37 weeks of gestation in relation to pregnancies ≥37 weeks of gestation) in singleton, twin and triplet pregnancies in Europe, 2006–2019**.

Data on premature deliveries were available from 21 countries (21 countries in 2018). Premature delivery rates (for fresh IVF and ICSI, FET, and ED together) according to multiplicity are presented in Supplementary Table S15. The incidence of extremely preterm birth (20–27 gestational weeks at delivery) was 1.5% in singleton pregnancies (1.0% in 2018), 3.3% in twins (3.1% in 2018), and 12.2% in triplets (6.0% in 2018). Very premature birth rates (28–32 gestational weeks at delivery) were recorded in 3.4% of singletons (2.2% in 2018), 10.9% of twin pregnancies (9.7% in 2018), and 40.8% in triplet pregnancies (37.9% in 2018). The evolution of the proportions of premature deliveries (before 37 weeks) over the years according to multiplicity is shown in Fig. 5. Term deliveries (≥37 weeks) were achieved in 84.2% (83.1% in 2018) of singleton pregnancies, 44.0% (43.6% in 2018) of twin pregnancies, and 8.2% (8.1% in 2018) of triplet pregnancies.

Complications and fetal reductions related to ART procedures were reported by 35 countries (34 in 2018) (Supplementary Table S16). The main reported complication was ovarian hyperstimulation syndrome (OHSS) (grades 3–5) with a total reported number of 1654 (1719 in 2018) corresponding to an incidence rate of 0.16% (0.17% in 2018). Other complications (1575; 1379 cases in 2018) were reported with a total incidence of 0.15% (0.14% in 2018) and with bleedings being the most frequent (0.1%, identical to 2018).

Two maternal deaths were reported (3 in 2018). France reported a 32-year-old patient who died 6 weeks after oocyte retrieval because of a massive pulmonary embolism. In Russia, a patient died after IVF because of a pulmonary embolism after severe OHSS in early pregnancy.

Fetal reductions were reported in 487 cases (509 in 2018), the majority from the UK, Belgium, and Ukraine.

### Preimplantation genetic testing

Table 1 includes PGT activities, which were reported from 27 countries (24 in 2018). The main contributors were Spain, Russia, Ukraine, Turkey, and Italy.

The total number of treatment cycles was 64 089 representing 6.9% of initiated IVF + ICSI and FET cycles together (48 294; 7.1% in 2018).

More details on PGT activities can be found in the annual reports of the ESHRE PGT Consortium (Spinella *et al.*, 2023).

### IVM

A total of 546 treatments with IVM were reported from nine countries (532 treatments from eight countries (Serbia) in 2018) (Table 1).

Most IVM cycles were recorded in Belgium, as in 2018. A total of 188 transfers resulted in 37 pregnancies (19.7% per transfer) and 25 deliveries (13.3% per transfer).

### Frozen oocyte replacement

A total number of 6299 thawing cycles were reported by 22 countries (5444 from 22 countries in 2018) (Table 1) with Italy and the UK being the largest contributors (1361 and 998 cycles, respectively).

Among 4402 transfers, 1075 resulted in pregnancies (29.5%; 29.5% in 2018) and 867 in deliveries (24.4%; 21% in 2018).

### IUI

Data on IUI-H or IUI-D were collected by a total of 1169 institutions (1271 in 2018) in 30 and 25 countries, respectively, as in 2018. Among 147 711 IUI-H (148 143 in 2018) and 51 651 IUI-D (50 609 in 2018) reported cycles, the numbers of IUI-H were the highest in France, Spain, Italy, Belgium, and Poland, and those of IUI-D were the highest in Spain, Belgium, Denmark, and the UK (Table 5 and Supplementary Tables S17 and S18).

**Table 5.** IUI with husband (IUI-H) or donor (IUI-D) semen in 2019.

	IUI-H						IUI-D					
Country	Cycles	Deliveries	Deliveries (%)	Singleton (%)	Twin (%)	Triplet (%)	Cycles	Deliveries	Deliveries (%)	Singleton (%)	Twin (%)	Triplet (%)
Albania	50	5	10.0	60.0	40.0	0.0						
Armenia	827	133	16.1	94.0	6.0	0.0	273	28	10.3	89.3	10.7	0.0
Austria	1846						517	0	0.0			
Belarus	1014	124	12.2	92.2	6.9	0.9	89	20	22.5	88.9	11.1	0.0
Belgium	12 293	789	6.4	94.4	5.6	0.0	9140	887	9.7	95.2	4.8	0.0
Bosnia-Herzegovina, Federation part	96	8	8.3	100.0	0.0	0.0						
Bulgaria												
Czech Republic												
Denmark	9504	1099	11.6	90.3	9.6	0.1	8514	554	6.5	94.4	5.6	0.0
Estonia	196	15	7.7	100.0	0.0	0.0	177	20	11.3	95.0	5.0	0.0
Finland	2561	241	9.4	93.4	6.6	0.0	1212	162	13.4	95.1	4.9	0.0
France	44 323	4762	10.7	90.3	9.3	0.3	2995	589	19.7	89.1	10.4	0.5
Germany												
Greece	3117	272	8.7	98.9	1.1	0.0	219	61	27.9	98.4	0.0	1.6
Hungary	2676	193	7.2	82.9	16.1	1.0						
Iceland	26	6	23.1	100.0	0.0	0.0	169	29	17.2	100.0	0.0	0.0
Ireland	434	62	14.3	87.1	12.9	0.0	182	30	16.5	92.3	7.7	0.0
Italy	15 895	1159	7.3	91.4	7.9	0.8	656	90	13.7	88.9	11.1	0.0
Kazakhstan	781	67	8.6	62.7	34.3	3.0	100	18	18.0	100.0	0.0	0.0
Latvia	96	5	5.2	100.0	0.0	0.0	39	4	10.3	75.0	25.0	0.0
Lithuania	737	50	6.8	93.8	6.3	0.0	7	1	14.3	100.0	0.0	0.0
Luxembourg	221	34	15.4	100.0	0.0	0.0	74	10	13.5	90.0	10.0	0.0
Malta	173											
Moldova	15	1	6.7	100.0	0.0	0.0						
Montenegro	273	14	5.1	71.4	28.6	0.0						
North Macedonia	1087	112	10.3	96.4	3.6	0.0	25	3	12.0	100.0	0.0	0.0
Norway	258	19	7.4	94.7	5.3	0.0	717	113	15.8	98.2	1.8	0.0
Poland	11 748	655	5.6	92.9	7.0	0.1	1673	194	11.6	96.9	3.1	0.0
Portugal	2115	192	9.1	93.2	6.3	0.5	491	78	15.9	92.2	6.5	1.3
Russia	7226	694	9.6	90.1	8.8	1.0	2813	351	12.5	94.2	5.8	0.0
Serbia	1346	25	1.9	88.0	12.0	0.0						
Slovakia	2107											
Slovenia	564	50	8.9	96.0	4.0	0.0						
Spain	18 984	1928	10.2	90.3	9.4	0.3	13 561	1948	14.4	92.4	7.2	0.4
Sweden							2310	323	14.0	98.1	1.9	0.0
Switzerland												
The Netherlands												
Turkey	1407	150	10.7	87.3	10.0	2.7						
Ukraine												
UK	3715						5698					
All^a^	147 711	12 864	9.2	90.9	8.7	0.4	51 651	5513	12.1	93.5	6.2	0.2

a Total refers to these countries where data were reported and mean percentage was computed on countries with complete information.

These data are an underestimation of the numbers, as IUI is not always part of the registry.

Delivery rates could be calculated for 139 870 IUI-H cycles (9.2%; 8.9% in 2018) and 45 436 for IUI-D cycles (12.1% versus 12.6% in 2018). Singleton deliveries were the most frequent regardless of the age group with an overall rate of 90.9% for IUI-H and 93.5% for IUI-D (91.2% in IUI-H, 93.4% in IUI-D in 2018). Twin and triplet rates were 8.7% and 0.4%, respectively, after IUI-H, and 6.2% and 0.2% after IUI-D, respectively (in 2018: 8.4% and 0.3%, respectively, after IUI-H and 6.4% and 0.2%, respectively, after IUI-D).

### Sum of fresh and FET (‘cumulative’) delivery rates

Supplementary Table S19 provides an estimate of a cumulative delivery rate. The cumulative delivery rate was calculated as the ratio between the total number of deliveries from fresh embryo transfers and FET performed during 1 year (numerator) and the number of aspirations during the same year (denominator). The cumulative delivery rate thus differs from a true cumulative delivery rate, which is based on all transfers resulting from one aspiration. The calculation was based on data provided by 37 countries (36 countries in 2018) with an overall delivery rate of 31.4% (32.3% in 2018). The cumulative increase resulting from additional FET (overall delivery rates from fresh embryo transfers) reached 15.3% (14.4% in 2018). In some countries, more deliveries were reported after FET than after a fresh IVF/ICSI cycle. Consequently, as a result of the relative contribution of FET the cumulative PR was high in Armenia, Belgium, Czech Republic, Denmark, Finland, Iceland, Ireland, Kazakhstan, Latvia, Malta, Moldova, Poland, Spain, Sweden, Switzerland, Turkey, and Ukraine. The relative lowest contribution of FET to the cumulative PRs came from Germany, Hungary, Lithuania, Malta, Montenegro, North Macedonia, and Serbia.

### Cross-border reproductive care

Thirteen countries reported data on cross-border reproductive care: Albania, Belarus, Czech Republic, Greece, Iceland, Ireland, Malta, Poland, Portugal, Slovenia, Spain, Switzerland, and Turkey. A total of 33 003 cycles (21 792 in 2018) were reported, 29.4% (21.5% in 2018) of which involved IVF/ICSI with the couple’s own gametes, 52.9% (52.6% in 2018) were oocyte reception cycles, and 13.7% (20.6% in 2018) were IVF or ICSI cycles with semen donation. Additionally, 2456 IUI with sperm donation (6791 in 2018) were registered. Information about the countries of origin was very incomplete and not reliable enough to draw any conclusion. The main reason reported by patients for crossing the borders was to seek less expensive treatment (43.0%; 25.0% in 2018). However, cross-border reproductive care was also reported to be performed because the treatment was of too low quality (29.7%; 42.3% in 2018) or not legal in the home country (13.8%; 21.1% in 2018).

### Fertility preservation

For the fourth year, data on FP were reported. Eighteen countries (16 in 2018 and 14 in 2017) provided data on a total number of 24 139 interventions (20 994 in 2018; 18 888 in 2017) (Supplementary Table S20) both for medical and non-medical reasons in pre- and post-pubertal patients. The majority of interventions consisted of the cryopreservation of ejaculated sperm (n = 11 592 with data from 16 countries, n = 10 503 with data from 14 countries in 2018) and the cryopreservation of oocytes (n = 10 784 with data from 15 countries, n = 9123 with data from 16 countries in 2018). Ovarian tissue cryopreservation was reported by 2 (2 in 2018) and 11 (11 in 2018) countries, respectively, for pre- and post-pubertal patients. The use of post-pubertal tissue through transplantation was reported by four countries (Greece, Italy, Slovenia, and Spain). Testicular tissue cryopreservation in post-pubertal patients and pre-pubertal boys was reported by 14 (8 in 2018) countries and by one country (1 in 2018), respectively.

## Discussion

Between 1997 and 2019, the EIM Consortium of ESHRE reported on a total of over 12 million treatment cycles (12 804 411) resulting in the birth of over 2 million infants.

The current 23rd annual report presents a comprehensive analysis of data on ART, IUI, and FP activities. The data are derived from compulsory or voluntary registries of 40 European countries (one more than in 2018). For the second time, the number of reported treatment cycles per year exceeded 1 million. Only a few countries opted out of participation (5 out of 44 EIM members as well as 7 non-EIM members including Azerbaijan, Kosovo, and 5 smaller countries not offering ART services). Furthermore, data could not be obtained from three member states of the EU (Croatia, Cyprus, and Romania), most probably because of economic, regulatory, or political factors, as suggested by a survey on medically assisted reproduction (MAR) activities (Calhaz-Jorge *et al.*, 2020).

Overall, the number of European countries actively participating has remained relatively stable in recent years, with only slight fluctuations. There has been a continued increase in the reported number of treatment cycles (+7.0% compared to 2018). In contrast, the number of infants born from ART per year declined (−5.4% compared to 2018), as did the percentage of ART infants per national births (3.0%; 3.5% in 2018).

Despite the well-known challenges associated with heterogeneous data collection systems in Europe and the lack of standardized indicators, the participation rate at the country level remains very high with 90.9% of EIM members contributing data after excluding those countries where ART services are not available. However, 21 countries (52.5% of EIM members) managed to submit data from all IVF institutions, resulting in 83.9% of all IVF institutions sending in their data (versus 91.6% in 2018). The main reason for this decrease is the newly installed participation of Turkey, where 26 out of 167 clinics (15.5%) submitted their data. Therefore, future efforts should prioritize the collection of complete data sets within each country.

To enhance the quality of the data from participating countries, progress is expected through implementing a prospective cycle-by-cycle data collection (already established in 16 countries in 2019) with harmonized indicators. As an initial step, a minimum core data set with defined outcome parameters and collected items was established (https://www.eshre.eu/Data-collection-and-research/Consortia/EIM).

Until better quality data are available, interpretation of the data should be carried out with caution. Better quality includes the harmonization of data collection systems across countries and registration of indicators, taking into account center/country-specific practices (e.g. freeze-all cycles, embryo transfer policy, PGT-A, etc.).

Besides the current EU objective to enhance vigilance in the field of MAR, increased transparency regarding access to reproductive care and cross-border treatments for all stakeholders is equally important. Over the years, the EIM Consortium has constantly recorded significant variation in access to treatment between countries, with the number of ART cycles per million women aged 15–45 years ranging from 3943 in Lithuania to 19 393 in the Czech Republic, and per million inhabitants from 694 in Lithuania to 3621 in the Czech Republic.

While such data are unique in Europe, the interpretation becomes more and more difficult owing to the historically estimated threshold of 1500 fresh ART cycles per million inhabitants. This threshold was previously considered necessary for adequate infertility care but technological advancements in the field have proved this outdated. Furthermore, cross-border patients also need to be considered when best estimates for sufficiency thresholds are established. Data on cross-border care were only available for 13 countries in 2019 (12 in 2018), indicating once more the need for a better pan-European registry.

Concerning treatment modalities, ICSI remains the most commonly used technique with a trend to stabilization of its use over the last few years (Table 1 and Fig. 1). FET is the second most employed technique. Over the years, higher proportions of FET treatment cycles [FET/(FET + ICSI + IVF)] were recorded and have now reached a relatively stable level (36.3% in 2019 and 35.5% in 2018). Higher proportions of FET treatment cycles were observed in other large registries (De Geyter *et al.*, 2020b). However, the proportion of FET cycles varies considerably among countries with complete data sets (ranging from 10.9% to 60.8%) highlighting the considerable variability in practices. This is also observed for freeze-all cycles (Supplementary Tables S5 and S6) reported since 2014, with an overall proportion of 12.7% (11.4% in 2018) per aspiration (oocytes and embryos together) for IVF and 17.3% (15.5% in 2018) for ICSI. However, these numbers also vary among countries with proportions reaching as high as 61.0% (29.8% in 2018) and 61.9% (41.7% in 2018) for IVF and ICSI, respectively. Initial studies showed that the freeze-all strategy could be beneficial for subgroups of patients; however, the policy is being more and more frequently used for all patient categories (Roque *et al.*, 2019).

This variability among countries should be considered when interpreting the data, especially regarding the evolution of pregnancy and delivery rates after fresh IVF and ICSI cycles (per aspiration) as well as after FET cycles (per thawing) over time (Table 3 and Figs 2A and B and 3A and B). Indeed, the higher success rates recorded with FET (per thawing) compared to fresh IVF and ICSI (per aspiration), presented here for comparison with previous reports, can be misleading. Several factors that may influence outcomes should be taken into account.

The PR per aspiration in IVF and ICSI seems to decrease over time. This can be seen in Fig. 2. Obviously, fresh cycles (per aspiration) can include cycles with no oocytes, cycles with failed fertilization, failed embryo development, and freeze-all cycles. Patients who benefit from embryo cryopreservation may even have a better prognosis.

It should also be noted that recorded delivery rates *per transfer* were comparable, but even higher after FET than after both IVF and ICSI cycles. One of the explanations could be that more blastocyst transfers were recorded in FET cycles (79%) as compared to blastocyst transfers in fresh IVF and ICSI combined (53%). Such observations, made in large data collection sets, play a crucial role in identifying research questions and potential causes, such as the influence of hormonal support on miscarriage rates or neonatal outcomes in FET cycles (Zaat *et al.*, 2021).

Cumulative delivery rates per cycle or per aspiration are better outcome indicators to assess treatment effectiveness (De Neubourg *et al.*, 2016). However, so far, the EIM consortium is unable to calculate true cumulative delivery and live birth rates since aggregated data are collected. As a result, the addition of outcomes from fresh and FET cycles within the same calendar year is used as a proxy indicator until a European cycle-by-cycle registry can be established (De Geyter *et al.*, 2023). When data from 37 countries (36 countries in 2018) were included, an approximated ‘cumulative’ delivery rate of 31.4% (32.3% in 2018) was recorded during the 1-year period. The additional benefit derived from FET cycles (compared to delivery rates from fresh embryo transfers) varied widely, ranging from 1.9% to 53.4%, reflecting most likely differences in freezing policies and indications.

Analyzing trends is important to inform the field about the adoption of data-driven approaches from registries and to assess subsequent modification of practices (Ferraretti *et al.*, 2017; De Geyter *et al.*, 2020a). For instance, the dissemination of EIM data sets increased awareness among professionals on the benefit of reducing the number of embryos replaced per transfer (Fig. 4A) to diminish multiple births (Fig. 4B). Consequently, most transfers now involve the replacement of a single embryo (elective or not) (55.4% of cycles versus 50.7% with single embryo replacement in 2018). Simultaneously, the proportion of both twin and triplet deliveries showed a small increase in 2019, although the overall trend shows a decrease (Fig. 4B). Twin and triplet rates for fresh IVF and ICSI cycles combined were 11.9% (range 0–26.9) and 0.3% (range: 0–3.8), respectively, while the corresponding results for FET were 8.9% and 0.1%.

In the future, it is expected that efforts will lead to the ultimate objective of achieving the birth of a single healthy child per embryo transfer and thereby reducing the risks associated with multiple births, such as prematurity (Fig. 5) (Land and Evers, 2003).

To promote singleton pregnancies through elective single embryo transfer and to reduce the time to pregnancy, embryo culture is often prolonged to the blastocyst stage. However, the benefit of blastocyst stage transfers on ART outcomes is still a matter of debate (Practice Committee of the American Society for Reproductive Medicine and Practice Committee of the Society for Assisted Reproductive Technology, 2018; Glujovsky *et al.*, 2022). In a multicenter randomized controlled trial in good prognosis IVF patients (≥4 available embryos), the live birth rate after fresh embryo transfer was higher in the blastocyst-stage group than in the cleavage-stage group (*P* = 0.035); however, a blastocyst-stage transfer policy did not, in this study, result in a significantly higher cumulative live birth rate compared to a cleavage-stage transfer policy (Cornelisse *et al.*, 2023).

When analyzing the developmental stage of the replaced embryos, the data showed that PRs for blastocyst transfers were higher compared to cleavage-stage embryos (39.4% versus 26.5%, respectively) in fresh IVF and ICSI cycles combined. In FET, the rates were 40.5% for blastocyst transfers versus 26.9% for cleavage-stage embryos. However, it is important to note that while blastocyst transfers result in higher pregnancy and live birth rates per transfer, they also result in lower numbers of embryos available for transfer. This highlights the importance of true cumulative outcome parameters. Unfortunately, the available data did not include the assessment of the time to pregnancy.

In addition to multiplicity and prematurity, other safety aspects of ART also remain underreported, among these the rate of complications for OHSS (0.16%, similar to 0.17% in 2018) and an incidence of all other complications being registered at 0.15% (0.14% in 2018). Reports on maternal deaths related to ART are even scarcer, with a best estimate of six maternal deaths per 100 000 IVF treatments directly related to IVF in a national cohort from The Netherlands, where OHSS and sepsis were the major causes (Braat *et al.*, 2010). It is noticeable that two maternal deaths after ART were registered in 2019 (Supplementary Table S16), both caused by pulmonary embolisms. At least one case was associated with OHSS.

Furthermore, while the age of recipients in ED cycles did not significantly affect the outcome of the cycle, risks associated with pregnancies in older women should not be overlooked as a potential safety aspect of the treatment. Indeed, a survey on the legislation and reimbursement aspects has shown that some countries do not have age limitations for recipients in ED cycles (Calhaz-Jorge *et al.*, 2020).

The crucial role of registries in MAR activities regarding outcome parameters and safety is well established (De Geyter *et al.*, 2016; Kissin *et al.*, 2019). The EIM data are also incorporated in the annual report of the worldwide IVF register from the International Committee for Monitoring Assisted Reproductive Technologies (Chambers *et al.*, 2021).

To enable reliable comparisons of practices and to identify the safest and most efficient care, it is essential to enhance the quality of collected data and strive to complete and harmonized data throughout Europe. Besides the establishment of clear definitions of registered items, providing the countries and competent authorities with an adapted IT solution should be the next priority. The European monitoring of Medically Assisted Reproduction (EuMAR) aims to develop a pan-European registry of prospective cycle-by-cycle data on the use and outcomes of MAR treatments (De Geyter *et al.*, 2023). EuMAR addresses the need for more transparency, surveillance, and biovigilance in MAR across country borders, including better data on the safety of MAR for offspring, donors, and recipients. These efforts align with the revision of the EU Directives on blood, tissues, and cells.

## Supplementary Material

dead197_Supplementary_Table_S1

dead197_Supplementary_Table_S2

dead197_Supplementary_Table_S3

dead197_Supplementary_Table_S4

dead197_Supplementary_Table_S5

dead197_Supplementary_Table_S6

dead197_Supplementary_Table_S7

dead197_Supplementary_Table_S8

dead197_Supplementary_Table_S9

dead197_Supplementary_Table_S10

dead197_Supplementary_Table_S11

dead197_Supplementary_Table_S12

dead197_Supplementary_Table_S13

dead197_Supplementary_Table_S14

dead197_Supplementary_Table_S15

dead197_Supplementary_Table_S16

dead197_Supplementary_Table_S17

dead197_Supplementary_Table_S18

dead197_Supplementary_Table_S19

dead197_Supplementary_Table_S20

dead197_Supplementary_Data_File_S1

## Data Availability

All data are incorporated into the article and its online supplementary material.

## Appendix

Contact persons who are collaborators and represent the data collection programs in participating European countries, 2019. All participating centers are listed in Supplementary Data File S1.

**Albania**

Prof. Orion Gliozheni, Department of Obstetrics & Gynecology, University Hospital for Obstetrics and Gynecology, Bul.B.Curri, Tirana, Albania. Tel: +355-4-222-36-32; Mob: +355-68-20-29-313; E-mail: glorion@abcom.al

**Armenia**

Mr Eduard Hambartsoumian, Fertility Center, IVF Unit, 4 Tigvan Nets, 375010 Yerevan, Armenia. Tel: +374-10-544368; E-mail: hambartsoumian@hotmail.com

**Austria**

Prof. Dr Heinz Strohmer, Dr Obruca & Dr Strohmer Partnerschaft Goldenes Kreuz-Kinderwunschzentrum, Lazarettgasse 16-18, 1090 Wien, Austria. Tel: +43-401-111-400; E-mail: heinz.strohmer@kinderwunschzentrum.at

**Belarus**

Dr Elena Petrovskaya (Alena Piatrouskaya), ART Centre “Embryo”, Filimonova 53, 220053 Minsk, Belarus. Tel: +375-293-830-570; E-mail: elenaembryoby@gmail.com

Dr Oleg Tishkevich, Centre For Assisted Reproduction “Embryo” Belivpul, Filimonova Str. 53, 220114 Minsk, Belarus. Tel: +375-296-222-722; Mob: +375-296-222-722; E-mail: tishol@tut.by

**Belgium**

Prof. Dr Diane De Neubourg, Antwerp University Hospital—UZA, Center for Reproductive Medicine, Drie Eikenstraat 655, 2650 Edegem, Belgium. Tel: +32-3-821-45-98; Mob: +32-475-69-91-18; E-mail: diane.deneubourg@uza.be

Dr Kris Bogaerts, I-Biostat, Kapucijnenvoer 35 bus 7001, 3000 Leuven, Belgium. Tel: +32-0-16-33-68-90; E-mail: kris.bogaerts@med.kuleuven.be

**Bosnia**

Prof. Dr Devleta Balic, Zavod za humanu reprodukciju “Dr Balic”, Kojsino 25, 75000 Tuzla, Bosnia, Herzegovina. Tel: +387-35-260-650; Mob: +387-611-402-22; E-mail: drbalic@bih.net.ba

**Bulgaria**

Irena Antonova, ESHRE certified clinical embryologist (2011), Ob/Gyn Hospital Dr Shechterev, 25-31, Hristo Blagoev Strasse, 1330 Sofia, Bulgaria. Tel: +359-887-127-651; E-mail: irendreaming@gmail.com

Dr Evelina Cvetkova, Executive Agency “Medical Supervision”, 3 Georgi Sofiyski Str, 1000 Sofia, Bulgaria. Tel: +359-879-011-601; E-mail: evelina.cvetkova@iamn.bg

**Czech Republic**

Dr Karel Rezabek, Department of Gynaecology, Obstetrics and Neonatology First Faculty of Medicine, Charles University and General University Hospital in Prague, Czech Republic, Apolinarska 18, 12000 Prague, Czech Republic. Tel: +420-224-967-479; Mob: +420-724-685-276; E-mail: rezabek.ivf@seznam.cz

**Denmark**

Dr John Kirk, Maigaard Fertilitetsklinik, Jens Baggensensvej 88 h, 8200 Arhus, Denmark. Tel: +45-86101388; Mob: +45-28696982; E-mail: john.kirk@dadlnet.dk

**Estonia**

Dr Deniss Sõritsa, Tartu University Hospital and Elitre Clinic, Tartu, Estonia. Tel: +372-740-9930; E-mail: soritsa@hotmail.com

**Finland**

Prof. Mika Gissler, THL National Institute for Health and Welfare, P.O. Box 30, 00271 Helsinki, Finland. Tel: +385-29-524-7279; E-mail: mika.gissler@thl.fi

Dr Sari Pelkonen, Department of Obstetrics and Gynaecology, Oulu University Hospital, P.O. Box 23, 90029 Oys, Finland. Tel: +358-8-3153040; E-mail: sari.pelkonen@fimnet.fi

**France**

Dr Imene Mansouri, Agence de La Biomedicine, 1, avenue du Stade de France, 93212 Saint-Denis la Plaine, France. Tel: +33-1-55-93-58-94; E-mail: imene.mansouri@biomedecine.fr

Prof. Jacques de Mouzon, 15-29 rue Guilleminot, 75014 Paris, France. Tel: +33-143-224-679; Mob: +33-662-062-274; E-mail: jacques.de.mouzon@gmail.com

**Germany**

Dr Andreas Tandler-Schneider, Fertility Center Berlin, Spandauer damm 130, 14050 Berlin, Germany. Tel: +49-30-233-20-81-10; E-mail: tandler-schneider@fertilitycenter-berlin.de

Dr Markus Kimmel, Deutsches IVF-Register e. V. (D·I·R), Lise-Meitner-Straße 14, 40591 Düsseldorf, Germany. Tel: +49-157-382-261-93; E-mail: geschaeftsstelle@deutsches-ivf-register.de

**Greece**

Prof. Nikos Vrachnis, National Authority of Medically Assisted Reproduction, Ploutarxou 3, P.O. 10675, Athens. Tel: +30-6974441144; E-mail: nvrachnis@hotmail.com

**Hungary**

Prof. Janos Urbancsek, First Department of Obstetrics & Gynecology, Semmelweis University, Baross utca 27, 1088 Budapest, Hungary. Tel: +36-1-266-01-15; E-mail: urbjan@noi1.sote.hu

Prof. G. Kosztolanyi, Department of Medical Genetics and Child Development, University of Pecs, Jozsef A.u, 7., 7623 Pecs, Hungary. Tel: +36-7-2535977; E-mail: gyorgy.kosztolanyi@aok.pte.hu

**Iceland**

Mr Hilmar Bjorgvinsson, IVF Klinikin Reykjavik, Alfheimum 74, 104 Reykjavik, Iceland. Tel: +354-430-4000; E-mail: hilmar.bjorgvinsson@ivfklinikin.is

**Ireland**

Prof. Mary Wingfield, Merrion Fertility Clinic, 60 Lower Mount Street, D02NH93 Dublin. Tel: +353-516635000; Mob: +353-872258556; E-mail: mwingfield@merrionfertility.ie

Ms Joyce Leyden, Merrion Fertility Clinic, 60 Lower Mount Street, D02NH93 Dublin. Tel: +353-516635000; Mob: +353-859290573; E-mail: jleyden@merrionfertility.ie

**Italy**

Dr Giulia Scaravelli, Istituto Superiore di Sanità, Registro Nazionale della Procreazione Medicalmente Assistita, CNESPS, Viale Regina Elena, 299, 00161 Roma. Tel: +39-06-499-04-050; E-mail: giulia.scaravelli@iss.it

Dr Roberto de Luca, Istituto Superiore di Sanità, Registro Nazionale della Procreazione Medicalmente Assistita, CNESPS, Viale Regina Elena, 299, 00161 Roma. Tel: +39-06-499-04-320; E-mail: roberto.deluca@iss.it

**Kazachtstan**

Prof. Dr Vyacheslav Lokshin, International Clinical Center for Reproductology “Persona”, Utepova Street 32a, 00506 Almaty, Kazakhstan. Tel: +7-727-382-7777; Mob: +7-701-755-8209; E-mail: v_lokshin@persona-ivf.kz

Dr Sholpan Karibayeva, International Clinical Center for Reproductology “Persona”, Utepova Street 32a, 00506 Almaty, Kazakhstan. Tel: +7-727-382-7777; E-mail: sh.karibaeva@gmail.com

**Latvia**

Dr Valerija Agloniete, Latvian Human Reproduction Society, Apuzes 14, 1046 Riga, Latvia. Tel: +371-29272497; E-mail: valerija.magomedova@gmail.com

**Lithuania**

Raminta Bausyte, Vilnius University Hospital Santaros Clinics, Santaros Fertility Center, Simono Staneviciaus 64-69, 07113 Vilnius, Lithuania. Tel: +370-620-86826; E-mail: raminta.bausyte@gmail.com

Ieva Masliukaite, Academic Medical Center, Center for Reproductive Medicine, Ijburglaan, 1086 ZJ Amsterdam, The Netherlands. Tel: +31-653-688-815; E-mail: i.masliukaite@amc.uva.nl

**Luxembourg**

Dr Caroline Schilling, Centre Hospitalier de Luxembourg, Centre de Stérilité et de Médecine de Reproduction, Rue Fiederspiel 2, 1512 Luxembourg, Luxembourg. Tel: +352-44-11-32-30; Mob: +352-66-13-13-912; E-mail: schilling.caroline@chl.lu

**Malta**

Dr Jean Calleja-Agius, University of Malta, 12, Mon Nid, Gianni Faure Street, TXN2421 Tarxien, Malta. Tel: +356-216-930-41; Mob: +356-995-536-53; E-mail: jean.calleja-agius@um.edu.mt

**Moldova**

Prof. Dr Veaceslav Moshin, Medical Director at Repromed Moldova, Center of Mother @ Child protection, State Medical and Pharmaceutical University, “N.Testemitanu”, Bd. Cuza Voda 29/1, Chisinau, Republic of Moldova. Tel: +373-22-263855; Mob: +373-69724433; E-mail: mosin@repromed.md; veaceslavmoshin@yahoo.com

**Montenegro**

Dr Tatjana Motrenko Simic, Human Reproduction Center Budva, Prvomajska 4, 85310 Budva, Montenegro. Tel: +382-33402432; Mob: +382-69-052-331; E-mail: motrenko@t-com.me

Dragana Vukicevic, Hospital “Danilo I”, Humana reprodukcija, Vuka Micunovica bb, 86000 Cetinje, Montenegro. Tel: +382-675-513-71; E-mail: vukicevic.dragana@yahoo.com

**The Netherlands**

Dr Jesper M.J. Smeenk, Department of Obstetrics and Gynaecology, St Elisabeth Hospital Tilburg, Hilv, The Netherlands. Tel: +31-13-539-31-08; Mob: +31-622-753-853; E-mail: j.smeenk@elisabeth.nl

**North Macedonia**

Mr Zoranco Petanovski, Hospital ReMedika, Nas. Zelezara, 1000 Skopje, Macedonia. Tel: +389-224-475-45; E-mail: zpetanovski@yahoo.com

**Norway**

Dr Liv Bente Romundstad, Spiren Fertility Clinic, Nardoskrenten 11, 7032 Trondheim, Norway. Tel: +47-73523000; Mob: +47-90550207; E-mail: libero@klinikkspiren.no

**Poland**

Dr Anna Janicka, VitroLive, Wojska Polskiego 103, 70-483 Szczecin, Poland. Tel: +48-91-88-69-260; E-mail: anna.janicka@vitrolive.pl

**Portugal**

Prof. Dr Carlos Calhaz-Jorge, CNPMA, assembleia da Republica, Palacio de Sao Bento, 1249-068 Lisboa, Portugal. Tel: +351-21-391-93-03; E-mail: calhazjorgec@gmail.com

Ms Joana Maria Mesquita Guimaraes, Hospital Geral Santo Antonio, Largo Professor Abel Salazar, 4050-011 Porto, Portugal. Tel: +351-96-616-02-37; E-mail: joanamesquitaguimaraes@gmail.com

Ms Patricia Duarte e Silva, CNPMA Avenida D. Carlos I, No. 134 4° Piso, 1200-651 Lisboa, Portugal; E-mail: patricia.silva@ar.parlamento.pt

**Russia**

Dr Vladislav Korsak, International Center for Reproductive Medicine, General Director, Liniya 11, Building 18B, Vasilievsky Island, 199034 St-Petersburg, Russia C.I.S. Tel: +7-812-328-2251; Mob: +7-921-9651977; E-mail: korsak@mcrm.ru

**Serbia**

Prof. Snezana Vidakovic, Institute for Obstetrics and Gynecology, Clinical Centre ‘GAK’, Visegradska 26, 11000 Belgrade, Serbia. Tel: +381-63-24-23-80; E-mail: drvidakovicsnezana@gmail.com

**Slovakia**

Dr Ladislav Marsik, Reprodulcia, Na križovatkách 58, 821 04 Bratislava. Tel: +421-905-251-904; E-mail: laco@marsik.sk

**Slovenia**

Prof. Borut Kovacic, Department of Reproductive Medicine and Gynecological Endocrinology, Univerzitetni klinicni center Maribor, Ljubljanska ulica 5, 2000 Maribor. Tel: +386-2-321-2160; Mob: +386-31-211-711; E-mail: borut.kov@ukc-mb.si

**Spain**

Mrs Irene Cuevas Saiz, Hospital General Universitario de Valencia, Avenida Tres Cruces, 2, 46014 Valencia, Spain. Tel: +34-963131800; Mob: +34-677245650; E-mail: cuevas_ire@gva.es

Dr Fernando Prados Mondéjar, Hospital de Madrid-Montepríncipe, HM Fertility Center Monteprincipe, C/Montepríncipe 25, 28660 Boadilla del Monte, Spain. Tel: +34-917-089-931; Mob: +34-646-737-237; E-mail: fernandojprados@gmail.com

**Sweden**

Prof. Christina Bergh, Department of Obstetrics and Gynaecology, Sahlgrenska University Hospital, Bla Straket 6, 413 45 Göteborg, Sweden. Tel: +46-31-3421000, +46-736-889325; Mob: +46-736-889325; E-mail: christina.bergh@vgregion.se

**Switzerland**

Ms Sandra Toitot, FIVNAT Office, c/o SGRM Geschäftsstelle, Postfach, 5001 Aarau 1, Switzerland. Tel: +41-62-836-20-90; E-mail: fivnat@fivnat-registry.ch

Dr Mischa Schneider, Kinderwunschzentrum Baden, Mellingerstrasse 207, 5405 Baden, Switzerland. Tel: +41-565001111; E-mail: schneidermischa@bluewin.ch

**Turkey**

Prof. Dr Mete Isikoglu, Gelecek Tup Bebek Merkezi, Caglayan Mh. Bulent Ecevit Bulvari 167, 07070 Antalya, Turkey. Tel: +90-554-2149493; Mob: +90-242324526; E-mail: misikoglu@hotmail.com

Dr Basak Balaban, Amerikan Hastanesi—American Hospital, Assisted Reproduction Unit, Güzelbahçe Sok, No. 20, Nisantesi, 34365 Istanbul, Turkey. Tel: +90-212-444-3-777; Mob: +90-532-253-27-92; E-mail: basakb@amerikanhastanesi.org

**Ukraine**

Prof Dr Mykola Gryshchenko, IVF Clinic Implant Ltd, Academician V.I.Gryshchenko Clinic for Reproductive Medicine, 25 Karl Marx Str., 61000 Kharkiv, Ukraine. Tel: +380-57-124522; Mob: +380-57-705070703; E-mail: nggryshchenko@gmail.com

**UK**

Dr Elliot Bridges, Human Fertilization and Embryology Authority (HFEA), 2 Redman Place, E201JQ, London EC1 Y 8HF, UK. Tel: +44-7817689677; E-mail: elliot.bridges@hfea.gov.uk

Dr Amanda Ewans, Human Fertilization and Embryology Authority (HFEA), 2 Redman Place, E201JQ, London EC1 Y 8HF, UK. Tel: +44-7724586896; E-mail: amanda.evans@hfea.gov.uk
