# Supplementary material for: ART in Europe, 2019: results generated from European registries by ESHRE
Source: Hum Reprod. 2023 Oct 17;38(12):2321–38. doi: 10.1093/humrep/dead197 (PMC10694409; doi:10.1093/humrep/dead197)
Supplement: dead197_Supplementary_Data_File_S1 [file dead197_supplementary_data_file_s1.doc]

**Supplementary Data File S1** Participating centers.

**Albania**

Tirana: Klinika Gliozheni

**Armenia**

Yerevan: Fertility Center; Vitromed; Shengavit; IVF Center of Institute of Perinatal Reproductive Medicine and Ob/Gyn; IVF Center of Institute of Maternal and child Health institute; IVF Center of Astkhik Medcial Center.

**Austria**

Baden: Wunschbabyinstitut Feichtinger

Bregenz: IVF-Zentren Prof. Zech

Dobl: Kinderwunschinstitut Schenk

Feldkirch: Kinderwunschzentrum Landeskrankenhaus Feldkirch

Graz: IVF Institut Dr. Steiner; Institut für Hormonstörungen Wechselbeschwerden und Kinderwunsch; Landeskrankenhaus Universitätsklinikum Graz

Innsbruck: Universitätsklinik Innsbruck; Private Kinderwunschklinik Dr. Josef Zech GmbH; WOMED Therapiezentrum Kinderwunsch GmbH

Klagenfurt: Vivaneo Kinderwunschklinik Sterignost

Krumpendorf: The Fertility Center Parkvilla

Linz: Kepler Universitätsklinikum GmbH – Med Campus IV,

Oberpullendorf: A. ö. Krankenhaus Oberpullendorf

Spital an der Drau: Kinderwunschinstitut Dr. Kaimbacher

St. Pölten: Tiny Feet Kinderwunschklinik

Salzburg: IVF-Zentren Prof. Zech Salzburg GmbH; Landeskrankenhaus Salzburg – Universitätsklinikum der PMU; Babywunsch-Klinik Dr. Zajc GmbH

Tulln: Kinderwunsch im Zentrum

Wien: Allgemeines Krankenhaus der Stadt Wien; Goldenes Kreuz; GYNANDRON Dr. Freude; Wunschbaby-Zentrum Feichtinger; Vivaneo Kinderwunschklinik; Die Wunschkind Klinik; Fertilitätszentrum Döbling

Wiener Neustadt: Tiny Feet Kinderwunschklinik

Wels-Thalheim: Vivaneo Kinderwunschklinik Dr. Loimer GmbH; IVF- und Kinderwunschinstitut Prof. Dr. Tews GmbH & Co KG

**Belarus**

Minsk: Center of assisted reproduction "Embryo"; GU Republican Scientific Practical Center “Mother and Child”; EVA CLINIC IVF; City Clinical Maternity Hospital #2; Multi-field Medical Company “LODE”

Gomel: Public health institution «Gomel Regional Diagnostic Center of Medical Genetics with consultation "Marriage and Family"

Vitebsk: Family Health Center “Bina”

**Belgium**

Antwerpen: ZNA Middelheim, Centrum voor Reproductieve Geneeskunde

Braine - L’alleud : C.H. Interregional Edith Cavell (CHIREC), Centre de Fécondation

Brugge: AZ Sint-Jan – Brugge-Oostende AV, CRG Brugge-Kortrijk

Brussel: UZ Brussel, Centrum voor Reproductieve Geneeskunde; Hôpital Universitaire Saint- Pierre - U.L.B., Clinique de Procréation Médicalement Assistée; Cliniques Universitaires Saint-Luc - U.C.L., Service de Gynécologie; Hôpital Erasme, Centre de FIV de l’U.L.B.

Charleroi : GHdC, Clinique Notre Dame, Service Gyn/Obst

Edegem: Universitair Ziekenhuis Antwerpen, Centrum voor Reproductieve Geneeskunde

Genk: Ziekenhuis Oost-Limburg - St. Jan, Genk Institute for Fertility Technology - GIFT

Gent: U.Z. – Gent, Vrouwenkliniek- afdeling Reproductieve Geneeskunde; A.Z. Jan Palfijn, Centrum voor Fertiliteitstherapie,

Leuven : Universitaire Ziekenhuizen K.U.Leuven Gasthuisberg, Dienst Gynaecologie; Regionaal Ziekenhuis Heilig Hart, Unit Reproductieve Geneeskunde

Libramont: Centre Hospitalier de l'Ardenne, Centre d’Infertilit,

Liege : Centre Hospitalier Régional de la Citadelle, Centre de FIV ; Clinic CHC MontLégia, Centre de Procréation Médicalement Assistée

Namur : Centre Hospitalier Régional de Namur, Service PM,

**Bosnia and Herzegovina**

Banja Luka: Medical center « Medico-S », member of Pronatal group, Republic of Srpka, BiH

Tuzla: BH IVF Centre « Dr Balic »

**Bulgaria**

Burgas: UMBAL “Burgas”
Pleven: Medical Center “KIRM”; Medical Center “Repromed”

Plovdiv: Medical Center “Bora”; USBALAG “Selena”; SMCG “New Life”

Sofia: SAGBAL “Dr Shterev”; MBAL Vita; Medical Center “Afrodita” ; IVF Clinic Medical Center “Dimitrov” ; Medical Center “Nadejda Reproductiv Sofia”; SBALGAR “Malinov”; Medical Center “Reprobiomed”; PSAGBAL “St Sofia”; SBALAGRM “Sofia”; SBALAG “Maichin Dom”; SBAL “St. Lazar”; Medical Center “Adela Fertility”; MBAL for women’s health “Nadejda”; Acibadem city clinic Tokuda, MBAL “Doverie”; Medical Center “Neo Vitro

Shumen: ASMP-MC “St. Ivan Rilski”

Stara Zagora: Medical Center “In vitro Trakia”

Tutrakan: Medical Center “St. Ivan Rilski”

Varna: AMCSMP “Maichin Dom”; MCAR “Varna”; MCRM “Nova radost”; ASMPMC “Olimed”

**Czech Republic**

Brno: Gyn. - por. klinika FN Brno-CAR01; MUDr. Aleš Bourek, PhD.; Reprofit International s.r.o.; Reprogenesis a.s Klinika reprodukční mediciny; Repromeda, s.r.o.; Sanatorium Helios, s.r.o.; Unica, s.r.o.

Ceské Budejovice: Pronatal Repro, s.r.o.; Sanatorium ART, s. r.o.

Hradec kralove: Sanus, s.r.o.

Jihlava: Sanus jihlava

Karlovy Vary: Institut reprodukční medicíny a genetiky, s.r.o.; Pronatal Spa, s.r.o. FertiCare SE Karlovy Vary

Kolin: Pronatal s.r.o.(Kolín)

Kostelec nad orlicí: Arleta ivf, s.r.o.

Liberec: Gennet s.r.o. liberec

Olomouc: Fakultni nemocnice Olomouc, CAR, Por.-gyn. Klinika; Fertimed, s.r.o.; IVF Clinic Olomouc

Ostrava: EuroFertil CZ,a.s.; Gyncentrum Ostrava, s.r.o., IVF Science Ostrava a.s.; Repromeda, s.r.o.

Pardubice: Sanus Pardubice

Plzen: FN Plzeň -CAR Gyn.-por.klinika; IVF - Zentren Prof. Zech - Pilsen s.r.o.; Natalart, s.r.o.

Praha: Europe IVF International ; Fakultní nemocnice v Motole – CAR- Gyn.- por. klinika 2. LF UK; FertiCare SE Praha; FertilityPort Prague s.r.o.; Gennet, s.r.o. Praha; Gennet,s.r.o.pobočka Archa; Gest, s.r.o.; Gynem Praha 8 ; Iscare I.V.F., a.s.; IVF Cube,s.r.o.; MMI Prague s.r.o.; Prague Fertility Centre s.r.o.; Pronatal Plus, s.r.o.; Pronatal, s.r.o.; Ustav pro péči o matku a dítě; Všeobecná fakultní nemocnice v Praze, CAR, Gyn.- por. Klinika 1. LF UK

Teplice: Pronatal Nord, s.r.o; Stellart s.r.o.

Zlín: IVF Czech Republic, s.r.o.

**Denmark**

Aalborg: Fertilitetsklinikken Aalborg Universitets Hospital;

Aarhus: Aagaard Fertilitetsklinik; Privathospitalet Ciconia; Maigaards Fertilitetsklinik

Copenhagen: Dansk Fertilitetsklinik; Copenhagen Fertility Center; Fertilitetsklinikken Herlev Hospital; Fertilitetsklinikken Hvidovre Hospital; Fertilitetsklinikken Rigshospitalet; Fertilitetsklinikken Trianglen; Junoklinikken; VitaNova; Stork IVFklinik

Fredericia: IVF-SYD

Horsens: Fertilitetsklinikken Regionshospitalet Horsens

Holbæk: Fertilitetsklinikken Holbæk Sygehus

Odense: Fertilitetsklinikken Odense Universitetshospital; Odense IVF-Klinik

Skive: Fertilitetsklinikken Skive Sygehus

**Estonia**

Tallinn: East Tallinn Central Hospital Women’s Clinic; Nova Vita Clinic; West Tallinn Central Hospital Women's Center for Reproductive Medicine; Fertility Clinic Nordic

Tartu: Tartu University Hospital's Women's Clinic; Clinic Elite

**Finland**

Helsinki: Dextra Helsinki; Felicitas Mehiläinen Helsinki; Ovumia Fertinova, Helsinki University Central Hospital

Joensuu: Northern Carelia Central Hospital

Jyväskylä: Ovumia Fertinova Jyväskylä

Kuopio: inOva; Kuopio University Central Hospital

Lappeenranta: Felicitas Mehiläinen Lappeenranta

Oulu: Felicitas Mehiläinen Oulu; Oulu University Central Hospital

Tampere: Ovumia Fertinova Tampere; Tampere University Central Hospital

Turku: Aura; Felicitas Mehiläinen Turku; Turku University Central Hospital

**France**

Aix en Provence : CH du pays d’Aix

Amiens: Groupe Santé Victor Pauchet

Angers: C.H.U. D’ Angers

Ars Laquenexy: CHR de Mercy

Avignon: Polyclinique Urbain V

Bagnolet: Centre médicochirurgical Floréal

Bayonne: Capio Clinique Belharra

Beaumont : Clinique La Chataigneraie

Besancon: CHU Jean Minjoz ; Polyclinique de Franche-Comté

Bezannes : CHI Nord Ardennes

Bondy : Hôpital Jean Verdier

Bordeaux : CHU Centre Aliénor d’Aquitaine

Brest : CHRU hôpital Morvan ; Clinique Pasteur Lanroze

Bron : Hôpital femme Mère Enfant

Bruges: Polyclinique Jean Villar

Caen: CHU de  Caen

Calais : CH de Calais

Chambray-Les-Tours: Pôle de Santé-Léonard de Vinci

Charleville-Mezieres: CHI Nord-Ardennes

Cherbourg-en-Cotentin : Polyclinique du COTENTIN

Clamart : APHP Hôpital Antoine Béclère

Clermont-Ferrand : CHU Estaing

Contamine-sur-arve: CH ALPES LEMAN

Créteil: CHI de Creteil

Dijon : Complexe Hospitalier du Bocage

Dreux : CH Dreux hôpital Victor Jousselin

Ecully : Clinique du Val d'ouest Vendome

Epinal : Polyclinique la ligne bleue

Ermont : Capio clinique Claude Bernard

Fort-de-France : Clinique Saint-Paul

Guilherand-Granges: Clinique Pasteur

La Roche sur Yon: Clinique Saint-Charles

La Tronche: Hôpital couple enfant

Le Blanc Mesnil: Hopital Privé De La Seine Saint Denis

Le Chesnay: Centre Medico Chirurgical de Parly II

Le Mans: Clinique du Tertre Rouge

Le Port: Clinique Jeanne d'Arc

Lens : CH Lens

Les Abymes : CHU de Pointe à Pitre Abymes

Lille : Hôpital Jeanne de Flandres ; hôpital prive Le Bois

Limoges : Hôpital de la mère et de l’enfant

Lorient : CH Bretagne Sud

Lyon: Clinic Natecia

Marseille: APHM Hôpital de la Conception ; Clinique Bouchard ; Hôpital St Joseph

Meaux : CH de Meaux

Montivilliers : Groupe hospitalier du Havre

Montpellier : CHU Hôpital Arnaud de Villeneuve ; Polyclinique Saint Roch

Mulhouse : Fondation du Diaconat

Nancy : Polyclinique Majorelle ; CHRU Nancy maternité

Nantes : Hôpital femme enfant adolescent ; Clinique Brétéché Viaud ; Clinique Jules Verne

Neuilly sur Seine: Hôpital Américain ; Centre Chirurgical Pierre Cherest

Nice: Clinique Saint Georges ; Hopital De L'archet

Nimes: CHU de Nimes  Hôpital Caremeau

Orleans: CHRO Hôpital de la source

Paris: Hopital Des Diaconesses; Hopital Pierre Rouques « Les Bluets » ; Hôpital Cochin-Hôtel Dieu-Broca ; Institut mutualiste Montsouris ; Clinique de La Muette ; APHP Hopital Bichat Claude Bernard ; APHP Hôpital Tenon

Pau: Polyclinique de Navarre

Perigueux: Clinique Francheville

Perpignan: Clinique Saint Pierre

Poissy: C.H. Int. De Poissy/St Germain en Laye

Poitiers: CHU de Poitiers

Puilboreau : clinique de l'Atlantique

Quint-Fonsegrives : Clinique Capio La Croix du Sud

Reims:  CHU de Reims Hopital Maison Blanche

Rennes: CHU de Rennes Hôpital Sud ; Clinique mutualiste La Sagesse

Roanne: CH De Roanne

Rouen: CHU de Rouen Hôpital Charles Nicolle ; Clinique Mathilde Rouen

Saint-Cloud: CH des Quatre villes site St Cloud

Saint-Denis: CH General Delafontaine

Saint-Herblain: Polyclinique de L’Atlantique

Saint-Martin-Boulogne : CMCO Côte D’Opale

Saint-Martin- d’Heres: Clinique Belledonne

Saint Pierre : Groupe Hospitalier Sud Réunion

Saint-Priest-en-Jarez : CHU de Saint Etienne Hôpital Nord

Salouel: CHU Amiens Sud

Saran : Polyclinique les longues allées

Schiltigheim : SIHCUS/CMCO

Senlis: GHPSO de Senlis

Suresnes: Hôpital Foch

Toulon : Clinique Saint Michel

Toulouse : Hôpitaux Mère & Enfants

Tours : C.H.R.U. de Tours hôpital Bretonneau

Valenciennes-Saint-Saulve : Centre d’AMP de la polyclinique du Parc

Villeurbanne: Médipole Lyon-Villeurbanne

Vitry Sur Seine: Hôpital Privé de Vitry Site Noriets

**Germany**

Aachen: Kinderwunschzentrum Aachen

Aalen: Kinderwunschzentrum Aalen

Amberg: Kinderwunschzentrum Amberg, Am Klinikum St Marien Amberg

Augsburg: Kinderwunschzentrum Augsburg, GMP

Bad Münder: Deutsche Klinik Bad Münder – Hannover, MVZ wagnerstibbe für Gynäkologie, Reproduktionsmedizin, Zytologie, Pathologie und Innere Medizin GmbH

Bad Schwartau: Kinderwunsch Holstein

Baden-Baden, ivf Baden-Baden GmbH

Bayreuth: MVZ Fertility Center Bayreuth GmbH

Berlin: Wunschkinder Berlin; Ceres – Kinderwunschzentrum Dr. Hannen und Dr. Stoll; MVZ Fertility Center Berlin, Auf dem Gelände der DRK Kliniken Westend; Kinderwunschärzte Berlin GbR, Zentrum für Kinderwunschbehandlung und Fertilitätsprotektion; Kinderwunschzentrum am Innsbrucker Platz Berlin; Kinderwunschzentrum am Potsdamer Platz; Kinderwunschzentrum an der Gedächtniskirche; Kinderwunschzentrum Dres. Hoffmann, Praxis-Klinik Dres. Hoffmann; Praxis für Kinderwunschtherapie, Berlin, Helle-Mitte; Praxis für Fertilität, Gynäkologische Endokrinologie und Reproduktionsmedizin;; MVZ TFP Berlin GmbH; MVZ Kinderwunschteam Berlin GmbH

Bielefeld: FROG – Kinderwunschinstitut und Frauenarztpraxis in der Praxisklinik Prof. Dr. med. Joachim Volz; Bielefeld Fertility-Center, Zentrum für Reproduktionsmedizin und Gynäkologische Endokrinologie

Bonn-Bad Godesberg, kiwup®: Kinderwunschpraxis in Bonn

Bonn: Kinderwunschzentrum Bonner Bogen; MVZ für Frauenheilkunde und IvF-Medizin Bonn GbR; Universitätsklinikum Bonn; Gynäkologische Endokrinologie und Reproduktionsmedizin

Bremen: Bremer Zentrum für Fortpflanzungsmedizin (BZF); Kinderwunsch Bremen

Chemnitz: Kinderwunschzentrum Praxisklinik City Leipzig, Dr. Gabert, Dr. Bauer, Dr. Schwandt (ÜBAG); Kinderwunschzentrum Leipzig-Chemnitz, Standort Chemnitz

Darmstadt: Kinderwunschzentrum Darmstadt

Deggendorf: Kinderwunschzentrum Niederbayern

Dortmund: Überörtliche Berufsausübungsgemeinschaft Kinderwunsch Dortmund, Siegen, Dorsten, Wuppertal GbR

Dresden: Gynäkologische Endokrinologie und Reproduktionsmedizin der Universitätsfrauenklinik Dresden, Universitäres Kinderwunschzentrum; Kinderwunschzentrum Dresden, Dr. med. univ. Birgit Leuchten

Düsseldorf: KinderwunschKö; MVZ TFP Düsseldorf GmbH; UniKiD, Universitäres interdisziplinäres Kinderwunschzentrum Düsseldorf

Erlangen: Kinderwunschzentrum Erlangen; Kinderwunsch Erlangen, Die Praxis für Reproduktionsmedizin; Universitätsklinikum Erlangen, Universitäts-Fortpflanzungszentrum Franken (UFF)

Essen: novum - Zentrum für Reproduktionsmedizin Essen- Duisburg, Überörtliche Gemeinschaftspraxis

Esslingen: IVF-Zentrum Esslingen

Frankfurt am Main: Gynäkologische Endokrinologie und Reproduktionsmedizin Re∙Pro∙Gyn, Universitätsklinikum Frankfurt am Main; Kinderwunsch & Hormonzentrum Frankfurt am Main, Am Palmengarten; repromedicum Kinderwunschzentrum; Reproduktionsmedizin - Gyn. Endokrinologie

Frechen-Königsdorf, Kinderwunschzentrum Königsdorf

Freiburg: CERF Centrum für Gynäkologische Endokrinologie & Reproduktionsmedizin Freiburg; Universitätsklinikum Freiburg; Klinik für Frauenheilkunde, Endokrinologie und Reproduktionsmedizin

Garching b. München: Kinderwunschpraxis München Nord

Gelsenkirchen: Kinderwunschpraxis Gelsenkirchen, Wissenschaftspark Pav. 8, 1.OG

Göttingen: MVZ Kinderwunschzentrum Göttingen; Zentrum für Kinderwunsch und Reproduktionsmedizin; gyn-medicum Göttingen

Grevenbroich: green-ivf; Grevenbroicher Endokrinologie- und IVF-Zentrum

Großhansdorf: Universitäres Kinderwunschzentrum Lübeck und Manhagen; Zentrum für Gynäkologische Endokrinologie und Reproduktionsmedizin am Universitätsklinikum Schleswig-Holstein, Universitäre Kinderwunschzentren GmbH, Standort Manhagen

Hagen: Freyja IVF Hagen; Kinderwunsch & Hormonzentrum

Halle (Saale): Universitätsklinikum Halle (Saale), Martin-Luther-Universität Halle-Wittenberg, Zentrum für Reproduktionsmedizin und Andrologie

Hamburg: amedes experts, Facharzt-Zentrum für Kinderwunsch, Pränatale Medizin, Endokrinologie und Osteologie Hamburg; Kinderwunsch Praxisklinik Fleetinsel Hamburg; Kinderwunsch Valentinshof; Kinderwunschzentrum Altonaer Straße (MVZ) im Gynäkologicum Hamburg (GbR); KinderwunschZentrum HAFENCITY Hamburg; MVZ Fertility Center Hamburg GmbH; Kinderwunsch Praxisklinik Fleetinsel Hamburg; Praxis für Kinderwunsch und Hormone

Hannover: Medizinische Hochschule Hannover (MHH), Abteilung Reproduktionsmedizin und gynäkologische Endokrinologie; Team Kinderwunsch Hannover

Heidelberg: Praxisgemeinschaft Kinderwunschzentrum Heidelberg; Universitäts-Frauenklinik Heidelberg, Abt. Gynäkologische Endokrinologie und Fertilitätsstörungen

Heinsberg, Kinderwunschzentrum Heinsberger Höfe GbR, Medizinische Kooperationsgemeinschaft

Hildesheim: Zentrum für Reproduktionsmedizin & Humangenetik; MVZ

Homburg: Klinik für Frauenheilkunde, Geburtshilfe und Reproduktionsmedizin, Universitätsklinikum des Saarlandes

Jena: Zentrum für Reproduktionsmedizin Jena & Erfurt, Gemeinschaftspraxis Dres. Friztsche

Kassel: MVZ für Reproduktionsmedizin am Klinikum Kassel

Kempten: KinderWunschKempten (KWK), Zentrum für gynäkologische Endokrinologie und Reproduktionsmedizin; Klinik für Frauenheilkunde und Geburtshilfe; Klinikum Kempten; Klinikverbund Allgaü

Kiel: fertilitycenter Schleswig-Holstein, fertilitycenterkiel / fertilitycenterflensburg; Kinderwunsch Kiel; Universitäres Kinderwunschzentrum Kiel

Köln: Klinik und Poliklinik für Frauenheilkunde und Geburtshilfe der Universität zu Köln, Gynäkologische Endokrinologie und Reproduktionsmedizin; MVZ amedes für IVF- und Pränatalmedizin in Köln GmbH; MVZ PAN Institut GmbH, Interdisziplinäres Kinderwunschzentrum

Langenhagen: Kinderwunschzentrum Langenhagen & Wolfsburg MVZ

Leer (Ostfriesland): Kinderwunschzentrum Ostfriesland

Leipzig: Kinderwunschzentrum Leipzig-Chemnitz, Standort Leipzig; Kinderwunschzentrum Praxisklinik City Leipzig, Dr. Gabert, Dr. Bauer, Dr. Schwandt (ÜBAG); Standort Leipzig

Lübeck: Universitäres Kinderwunschzentrum Lübeck, Zentrum für Gynäkologische Endokrinologie und Reproduktionsmedizin am Universitätsklinikum Schleswig-Holstein, Universitäre Kinderwunschzentren GmbH

Ludwigsburg: Kinderwunschzentrum Ludwigsburg

Ludwigshafen: Kinderwunschzentrum Ludwigshafen

Magdeburg: Kinderwunschzentrum Magdeburg; Otto-von-Guericke-Universität Magdeburg, Universitätsfrauenklinik, Bereich Reproduktionsmedizin und Gynäkologische Endokrinologie

Mainz: Kinderwunsch Zentrum Mainz; Kinderwunschzentrum der Universitätsmedizin Mainz

Mannheim: Kinderwunschzentrum der Universitätsmedizin Mannheim

Marburg: RepKo - Reproduktionsmedizinisches Kompetenzzentrum am Universitätsklinikum Gießen & Marburg GmbH

Minden: Zentrum für Kinderwunschbehandlung und pränatale Medizin; GMP

Mönchengladbach: Ki.Nd, Kinderwunschzentrum Niederrhein

München: Kinderwunschzentrum A.R.T. Bogenhausen, MVZ für gynäkologische Endokrinologie und Reproduktionsmedizin, Akademische Lehrpraxis der TU München; Hormon- und Kinderwunschzentrum; Klinik und Poliklinik für Frauenheilkunde und Geburtshilfe der LMU München; Kinderwunsch Centrum München; kïz) kinderwunsch im zentrum, Praxis für gynäkologische Endokrinologie und Reproduktionsmedizin; Reproduktionsmedizin München im Tal, MVZ Partnergesellschaft mbH; Zentrum für Gynäkologische Endokrinologie und Reproduktionsmedizin, Klinikum der LMU München-Innenstadt

Münster: Kinderwunschpraxis an der Promenade, GMP Mempel & Stratmann; MVZ Kinderwunsch und Hormonzentrum Münster GmbH, Kinderwunschzentrum Münster; UKM Kinderwunschzentrum, Universitätsklinikum Münster

Neckarsulm: KinderwunschFrauenaerzte, Kinderwunschzentrum Neckarsulm

Neuwied: Kinderwunschzentrum Mittelrhein

Nürnberg: Kinderwunsch und Frauen-Hormon Centrum Nürnberg

Offenbach: MVZ Kinderwunsch- und Endometriosezentrum Offenbach

Oldenburg: Tagesklinik Oldenburg; Team Kinderwunsch Oldenburg

Osnabrück: Zentrum für Kinderwunschbehandlung Osnabrück

Pforzheim: Centrum für Kinderwunsch Pforzheim

Potsdam: Kinderwunschzentrum Potsdam MVZ GmbH

Prien am Chiemsee: Kinderwunsch Centrum Chiemsee

Recklinghausen: REProVita; Kinderwunschzentrum Recklinghausen

Regensburg: MVZ KITZ Regensburg GmbH; profertilita ; Fachklink für Fruchtbarkeitsmedizin

Remscheid: Bergisches Kinderwunschzentrum Remscheid

Rostock: Praxis für Fertilität; MVZ GmbH

Saarbrücken: IVF-SAAR Saarbrücken-Kaiserslautern

Singen: Kinderwunsch Bodensee

Stuttgart / Bad-Cannstatt, Kinderwunschzentrum Bad Cannstatt, Praxis M. Woriedh

Stuttgart: Kinderwunsch-Zentrum Stuttgart, Praxis Villa Haag; Kinderwunschärzte Stuttgart, Überörtliche Berufsausübungsgemeinschaft Dr. med. Tekesin & Dr. med. Kircher GbR; Next Fertility Stuttgart, Zweigniederlassung der MVZ Next Fertility Ulm GmbH

Trier: Kinderwunsch Praxisklinik Trier; Wissenschaftspark (WIP)

Tübingen: IVF-Zentrum der Universitäts-Frauenklinik Tübingen; KinderwunschPraxis Dres. Göhring

Ulm: MVZ Next Fertility Ulm GmbH; Universitätsfrauenklinik Ulm, UniFee – Kinderwunsch / Fertility and Endocrinology

Viernheim: Viernheimer Institut für Fertilität

Wetzlar: Kinderwunschzentrum Mittelhessen

Wiedbaden: MVZ TFP Rhein-Main GmbH

Würzburg : MainKid, Kinderwunschzentrum am Theater; Universitätsklinikum Würzburg, Frauenklinik und Poliklinik, Zentrum für gynäkologische Endokrinologie und Reproduktionsmedizin (ZERM); Zentrum für Reproduktionsmedizin und Pränataldiagnostik

**Greece**

Attiki - Athens: University Hospital Aretaieion, IVF unit; Embryogenesis IVF unit; Eugonia IVF unit ; Akeso IVF UNIT; IVF Athens Center; Gennima IVF unit; Neogenesis IVF unit; Embryoart IVF unit; Genesis Athens, IVF unit; Embryoland IVF unit; Iakentro Athens; Iaso Athens, IVF unit; Biodimiourgia, IVF Unit; Institouto Gonimothtas; Mitosis IVF unit; Kapetanakis IVF Center; Kentro Biogenetikhs; Medimall IVF Unit; Monada Anaparagogikis Iatrikis IVF Unit; Mitrotis IVF UNIT; Assisted Reproduction Unit; emBIO IVF unit; Maternity Health Unit; General Hospital Athens ALEXANDRA; University Hospital Attikon, IVF Unit; Mitera Hospital, IVF Unit;  Rea Maternity, IVF unit; Serum IVF Unit; University hospital Elena Benizelou; Titani Ziogas Vasileios IVF Unit

Crete: Mediterranean Fertility Institute  IVF UNIT Crete; Crete Fertility Centre ivf unit

Ioannina: University Hospital Ioanninon, IVF unit

Patra: Genesis Fertility centre

Thessaloniki: University Hospital Papageorgiou IVF unit; Thessaloniki IVF center; Iakentro Thessaloniki;  Assisting Nature IVF Unit; Biogenesis IVF Unit;  Embryoclinic, IVF unit; Embryolab, IVF unit;  Newlife SA, IVF unit; Genesis Fertility IVF unit; Fertility Clinic; Interbalkan medical centre

Thessaly: University Hospital Thessalias, IVF Unit; Iaso Thessalias, IVF unit

Thrace: Embryokosmogenesis IVF Unit

**Hungary**

Budapest: Division of Assisted Reproduction, Dept. of OB/GYN, Semmelweis University, Faculty of Medicine; Buda Infertility Center, St. John’s Hospital; Sterility Devai Institute; Forgacs Institute; Róbert Károly Infertility Center; Kaáli Institute; Versys Clinics Human Reproduction Institute; Reprosys Reproduction Center

Debrecen: Assisted Reproduction Center, University of Debrecen

Gyõr: Kaáli Institute

Kaposvár: Kaposi Mór Teaching Hospital

Pécs: Departement of OB/GYN, University of Pécs

Szeged: Kaáli Institute, Pannon Reproduction Institute

Tapolca: Kaáli Institute

**Iceland**

Reykjavik: Livio Reykjavik

**Ireland**

Cork:     Waterstone Clinic

Dublin: Femplus Women’s Health Clinic

Dublin: Merrion Fertility Clinic

**Italy**

Acerra: Villa dei Fiori s.r.l.

Ancona: Centro PMA - Presidio Ospedaliero “G. Salesi”.

Aosta: Centro PMA Valle d’Aosta.

Appiano Gentile: Le Bettulle Casa di Cura s.r.l. - Unità di Procreazione Medico Assistita

Arco: Centro Provinciale per la Procreazione Medicalmente Assistita - Ospedale Alto Garda e Ledro.

Asti: Centro di Medicina della Riproduzione - SOC Ostetricia e Ginecologia dell'Ospedale Cardinal Massaia.

Avellino: Fisiopatologia della Riproduzione e Sterilità di Coppia - A.O.R.N. San Giuseppe Moscati di Avellino.

Bari: Centro Medico "San Luca"; Centro PMA - Casa di Cura Santa Maria.

Barletta: Pro Andros s.r.l.

Battipaglia: Embryos S.r.l.

Benevento: Centro di Fisiopatologia della Riproduzione Umana - AORN "G. Rummo" di Benevento.

Bergamo: Centro di Fisiopatologia della Riproduzione - A.O. Papa Giovanni XXIII.

Bisceglie: MOMO' FertiLIFE.

Bologna: SISMER - Società Italiana Studio Med. Ripr.; Str. Sempl. Dipt. di Infertilità e PMA - Centro di riferimento regionale per la preservazione della fertilità nei pazienti oncologici - Dipt. Attività Integrata del Bambino, della Donna e delle Malattie Urologiche - AOU Policlinico S. Orsola Malpighi; Tecnobios Procreazione s.r.l.; Poliambulatorio Privato Day Surgery Next Fertility GynePro Medical.

Bolzano: Donna Salus.

Bra: Centro PMA - Casa di Cura "Città di Bra".

Brescia: Centro di Fecondazione Medicalmente Assistita - U.O. Ostetricia e Ginecologia - Casa di Cura "Istituto Clinico Città di Brescia"

Brindisi: Progenia S.r.l.; Casa di Cura SALUS s.r.l.

Brunico: Centro di medicina della riproduzione umana e crioconservazione gameti - Ospedale di Brunico.

Cagliari: Centro per la Diagnosi e Cura della sterilità di Coppia - Università degli Studi di Cagliari - Ospedale S. Giovanni di Dio di Cagliari (now closed); Servizio Ostetricia e Ginecologia - Diagnosi Prenatale e Preimpianto - Opedale Regionale Microcitemico di Cagliari.

Cantù: Centro di Fisiopatologia della Riproduzione del P.O. di Cantù - ASST LARIANA.

Carmagnola: Policlinico San Luca s.r.l.

Caserta: Genesis Day Surgery & Scientific Research s.c.a.r.l.; CARAN srl - Medicina e Biologia della Riproduzione.

Cassino: Centro PMA del Cassinate.

Catania: Azienda Ospedaliera Universitaria Vittorio Emanuele - Presidio Ospedaliero Santo Bambino - Centro di Fisiopatologia della Riproduzione Umana; C.R.A. Centro di Riproduzione Assistita s.r.l.; Centro di Medicina della Riproduzione; Centro di Ginecologia e Medicina della Riproduzione GmR di Giuseppe Iraci Sareri; Casa di Cura Falcidia srl; A.O. "Cannizzaro" - Centro di PMA; Arnas Garibaldi - Centro di PMA P.O. Garibaldi di Nesima.

Catanzaro: Servizio di Procreazione Medicalmente Assistita dell'A.O. Pugliese-Ciaccio.

Cattolica: U.O. Fisiopatologia della riproduzione umana - Ospedale Cervesi di Cattolica - Azienda USL della Romagna; Extra Omnes - Medicina e Salute Riproduttiva.

Cava de’ Tirreni: Artemisia H. S.r.l. - Casa di Cura R. Ruggiero;

Chianciano Terme: Chianciano Salute - Centro di Chirurgia Ambulatoriale.

Chieti: Casa di Cura Spatocco - Centro I.E.M.A. - Istituto Europeo Medicina della Riproduzione Abruzzese.

Città Sant’Angelo: Casa di Cura Villa Serena del Dott. L. Petruzzi s.r.l.

Cittadella: Centro di Fecondazione Medicalmente Assistita di Cittadella - U.O.A. Ostetricia e Ginecologia - Dipartimento Materno Infantile - P.O. di Cittadella.

Conegliano: Centro Regionale Specializzato di Fisiopatologia della Riproduzione - Ospedale Civile di Conegliano.

Conversano: U.O. Fisiopatologia della Riproduzione Umana e P.M.A.

Cortona: Centro Procreazione Medicalmente Assistita - Ospedale Valdichiana Santa Margherita.

Erice: Casa di Cura Sant'Anna Centro di PMA (now closed).

Fermo: Casa di Cura Palmatea - Centro satellite SISMER.

Firenze: Florence - Centro di Chirurgia Ambulatoriale ed Infertilità; SOD di Procreazione Medicalmente Assistita - Università degli Studi di Firenze - A.O. Careggi; Centro di Procreazione Assistita "Demetra"; Futura Diagnostica Medica - Procreazione Medicalmente Assistita s.r.l.

Forlì: Centro di Procreazione Medico Assistita Villa Serena.

Fossano: S.S. Fisiopatologia della Riproduzione Umana.

Frosinone: Centro Medico Life Srl.

Galliate: Centro Procreazione Assistita c/o Sede di Galliate.

Genova: Medicina della Riproduzione - Ospedale Evangelico Internazionale; UOS Fisiopatologia della Riproduzione Umana IRCCS Policlinico Ospedale San Martino Genova.

Gioia Tauro: Gatjc - Gioia Tauro

Giugliano In Campania: Clinic Center HERA – Centro HERA srl.

Gragnano: Studio A.G.O.I. del Dott. A. M. Irollo

Grosseto: Demetra - Centro Grossetano per la Cura dell'Infertilita'

Lagosanto : Fisiopatologia della riproduzione umana - Ospedale del Delta.

Lamezia Terme: C.I.S. Medicina Della Riproduzione

L'Aquila: U.O.C. Ostetricia e Ginecologia D.U. (con Centro Fivet) - del P.O. dell'Aquila.

Latina: Ospedale Santa Maria Goretti - Dipartimento Materno lnfantile - UOC Ostetricia e Ginecologla DEA ll - UOS Fisiopatologia della Riproduzione (PMA).

Lecce: Casa di Cura “Prof Petrucciani” – Centro di Procreazione Medicalmente Assistita

Livorno: CRPO - Medicina della Riproduzione.

Lugo: Servizio di Fisiopatologia della Riproduzione Umana - AUSL della Romagna P.O. di Lugo.

Maddaloni: IATREION s.r.l.- Medicina Polispecialistica

Manerbio: Dipartimento di PMA - U.O. Ostetricia e Ginecologia - A.O. di Desenzano del Garda - P.O. di Manerbio.

Mantova: Centro di Medicina della Riproduzione ed Endocrinologia - U.O. Ostetricia e Ginecologia - A.O. “Carlo Poma”.

Marcianise: UOSD di Fisiopatologia della Riproduzione-P.O. di Marcianise- ASL Caserta

Marostica: Genera Veneto Srl.

Martellago: GENESI S.r.l.

Mazara Del Vallo: Terzo Millenio s.r.l.

Merano: EuBios - Centri Fivet Prof. Zech.

Messina: Centro Riproduzione Umana CRU s.r.l.; Azienda Ospedaliera Papardo - Centro PMA

Milano: PMA Fondazione Ca’ Granda Ospedale Maggiore Policlinico U.O. Semplice Dipartimentale (UOSD) - PMA; Centro Scienze della Natalità - I.R.C.C.S. Ospedale San Raffaele; ESPA - Centro Endocrinologia, Sterilità e PMA - Ospedale M. Melloni - A.O. Fatebenefratelli e Oftalmico - U.O. Ostretricia e Ginecologia; Centro di Fecondazione Assistita - Casa di Cura "La Madonnina S.P.A."; Casa di Cura IGEA - Servizio Procreazione Medicalmente Assistita; Centro Studi e Trattamento per Disturbi della Fertilità - U.O. Ostetricia e Ginecologia - U.O. Ospedale Niguarda Cà Granda; Centro di Procreazione Medico Assistita - Istituto Clinico Città Studi SpA; Procreazione Medicalmente Assistita - PMA - A. O. "San Paolo" di Milano; Centro di Riproduzione Assitita - U.O. Ostetricia e Ginecologia - A.O. Luigi Sacco - P.O. Luigi Sacco; Centro PMA - Istituto Clinico Sant'Ambrogio.

Modena: Centro di Medicina della Riproduzione - Dip.to Scienze Mediche e Chirurgiche Materno-Infantili e dell'adulto - Sez. di Ginecologia ed Ostetricia - A.0. Università di Modena Policlinico; Clinica Eugin.

Molfetta: Prolab srl.

Montauro: Centro di Medicina della Riproduzione "Diagnostica Nausicaa s.r.l.".

Montichiari: Centro di Procreazione Assistita - U.O. Ostetricia e Ginecologia - A.O. "Spedali Civili" di Brescia - P.O. di Montichiari.

Monza: Centro di Medicina della Riproduzione BIOGENESI- Istituti Clinici Zucchi.

Napoli: Centro di Sterilità ed Infertilità - Ospedale Internazionale Casa di Cura s.r.l. (now closed); Centro di PMA - Clinica Mediterranea; Centro di Fecondazione Assistita Villa Bianca; Centro PMA del Dipartimento Assistenziale di Ostetricia, Ginecologia e Neonatologia - A.O.U. Policlinico - Università Luigi Vanvitelli; Centro di Sterilità - Az. Univ. Policlinico - Università degli Studi di Napoli Federico II; Centro di Procreazione Medicalmente Assistita - Casa di Cura Ruesch; Global life srl - Centro Fecondazione Assistita; Medicina della Riproduzione P.O. San Paolo.

Nardò: U.O. di Fisiopatologia della Riproduzione e Centro PMA - Ospedale di Nardò.

Oderzo: Centro per la Procreazione Medicalmente Assistita "Gianluigi Beltrame" - Ospedale di Oderzo, ULSS9 Treviso.

Ortona: Centro di Procreazione Medicalmente Assistita - ASL n° 2 Università degli studi "G. d’Annunzio" Chieti Pescara - Ospedale Civile "G. Bernabeo" di Ortona

Osio Sotto: Centro di PMA Istituti Ospedalieri Bergamaschi - Policlinico San Marco.

Paderno Dugnano: Clinica San Carlo - Casa di Cura Privata Polispecialistica Spa U.O. Ostetricia e Ginecologia - Procreazione Medicalmente Assistita.

Padova: Diagnostica Riviera srl; Unità Operativa Semplice Procreazione Medicalmente Assistita - Università - Azienda di Padova; Biotech PMA s.r.l.

Palermo: Genesy s.r.l. Contratto c/o Casa di Cura Serena s.p.a.; Centro Andros s.r.l.; Procreazioni Assistite Demetra (now closed); A.M.B.R.A. Associazione Medici e Biologi per la Riproduzione Assistita; Centro di Biologia della Riproduzione C.B.R; Casa di Cure Cosentino s.r.l. "Servizio di Fisiopatologia della Riproduzione Umana IVF Mediterranean Centre" (now closed); Ginecon (now closed); Centro Interaziendale di PMA - AOOR Villa Sofia – Cervello (now closed).

Parma: Centro procreazione medicalmente assistita (CPMA) - Clinica Ostetrica e Ginecologica - Azienda Ospedaliero - Universitaria di Parma (now closed).

Pavia: IRCCS Policlinico San Matteo di Pavia - 1 - Clinica Ostetricia e Ginecologia - Centro Procreazione Medicalmente Assistita.

Pesaro: Centro di Medicina della Riproduzione e Tecniche di Fecondazione Assistita - UOC Ostetricia e Ginecologia - Az. Osp. Ospedali Riuniti Marche NORD.

Piacenza: Inacqua Centro Medico (now closed).

Pieve Di Cadore: Ospedale "Giovanni Paolo II" Pieve di Cadore.

Pisa: Fertility IVF Unit "Pina de Luca" c/o Casa di Cura San Rossore; Percorso Infertilità e Procreazione medicalmente assistita. Azienda Ospedaliero Universitaria Pisana; Centro Medico Esculapio.

Pompei: MEDICA FUTURA srl.

Ponsacco: Centro PMA Valdera.

Ponte San Pietro: Centro PMA - Policlinico San Pietro.

Pordenone: S.S.D di Procreazione Medicalmente Assistita (now closed).

Potenza: Azienda Ospedaliera Regionale "San Carlo" - Dipartimento della donna e del bambino - U.O.C. Ostetricia e Ginecologia - Procreazione Medicalmente Assistita.

Pozzuoli: Centro di PMA - P.O. Santa Maria delle Grazie di Pozzuoli - ASL NA2 nord

Ragusa: "Medi.San S.r.l." c/o Casa di Cura del Mediterraneo.

Reggio Emilia: Centro per la Diagnosi e la Terapia della Sterilità Involontaria di Coppia "P-Bertocchi"- Arcispedale S. Maria Nuova AUSL di Reggio Emilia; Centro Palmer srl (now closed).

Rende: LIFE LAB - Studio Medico Specialistico di Riproduzione Medicalmente Assistita e Andrologia.

Rimini: Nuova Ricerca s.r.l.

Roma: Casa di Cura Privata European Hospital; RAPRUI s.r.l.; Casa di Cura privata Villa Salaria; One Day Medical Center; Centro della Salute e Tutela della Donna e del Bambino Sant'Anna; C.I.P.A. Centro Italiano Procreazione Assistita - Studio di Diagnosi Medica S.r.l.; Centro LEDA di Najjar Robert; UOC SMU CO3 Fisiopatologia della Riproduzione - DAI Materno Infantile e Scienze UroGinecologiche - AOU Policlinico Umberto I; ARTEMISIA SpA; Centro Biofertility; Casa di cura privata Villa Mafalda; Casa di cura "Villa Margherita"; Centro di Sterilità - Istituto di Ginecologia - Policlinico A. Gemelli - Università Cattolica del Sacro Cuore; Centro di Procreazione Medicalmente Assistita - A.O. "San Filippo Neri"; UOC Fisiologia della Riproduzione - Presidio Ospedaliero Sandro Pertini; Praxi ProVita; Alma Res; Grimaldi Medical S.r.l.; Fisiopatologia della Riproduzione Umana - U.O.di Ginecologia e Ostetricia - Azienda Ospedaliera S. Camillo – Forlanini (now closed); Diagnostica Fabia Mater; Clinica Valle Giulia Casa di Cura Spa; Centro PMA Villa Pia; Casa di Cura Nuova Villa Claudia; IVI Roma Casilino.

Rozzano: I.R.C.C.S. Istituto Clinico Humanitas - Dipartimento di Ginecologia e Medicina della Riproduzione.

Salerno: Fertilitas M.R.D.S. (Medicina della Riproduzione Day Surgery) s.r.l.; GEA Medicina della Riproduzione del Dott. Mario Cirmeni (now closed); Mediterraneo Medicina della Riproduzione; Casa di Cura Tortorella S.p.a.

San Donà di Piave: Centro Medicina S.P.A. - Filiale di San Donà di Piave.

San Gennaro Vesuviano: Casa di Cura "La Madonnina" s.r.l.

San Giorgio a Cremano: Centro Fecondazione Assistita "Andrea Grimaldi".

Sant’Agata Li Battiati: Centro di Procreazione Medicalmente Assistita U.M.R.

Santorso: Centro di Procreazione Medico - Assistita Ospedale di Santorso.

Sassari: Centro per PMA - Clinica Ostetrica e Ginecologica dell'Università di Sassari - A.O.U. di Sassari.

Siena: Centro Diagnosi e Cura Sterilità - Università degli Studi di Siena - U.O. Ostetricia e Ginecologia - Policlinico Le Scotte - P.O. Senese; A.G.I. Medica PMA.

Sora: Centro S.T.S. s.r.l. - Ambulatorio Fisiopatologia della Riproduzione - Ginecologia ed Endocrinologia.

Taranto: CREA s.r.l.

Todi: Centro di Procreazione Medicalmente Assistita Clinica Ostetrica e Ginecologica Azienda Ospedaliera di Perugia Ospedale della Media Valle del Tevere – Pantalla.

Torino: Centro Clinico San Carlo di Fecondazione Assistita e Ginecologia; Livet s.r.l.; Promea s.p.a.; C. M. R. Centro di Medicina Riproduttiva e Procreazione Assistita; Centro Medicina della Riproduzione - AOU città della Salute e della Scienza di Torino - Ospedale Sant'Anna; Centro Fivet Città di Torino - c/o Casa della Salute ex Ospedale di Valdese; Sedes Sapientae.

Tortona: Medicart - Centro Cardiologico e Polispecialistico s.r.l.

Trecenta: Centro PMA - Presidio Ospedaliero S. Luca – Trecenta – ULSS 5 Polesana.

Trieste: SCR Fisiopatologia della Riproduzione e Procreazione Medicalmente Assistita - IRCCS Burlo Garofolo.

Udine: Policlinico Città di Udine SpA - Casa di Cura Privata.

Umbertide: Ambulatorio Chirurgico di Ginecologia ed Ostetricia - Genera Umbria s.r.l.

Vallo Della Lucania: Centro di PMA - U.O. Ostetricia e Ginecologia - P.O. San Luca di Vallo della Lucania.

Varese: ASST Settelaghi - c/o UO di Ostetricia e Ginecologia del Presidio Ospedaliero Filippo del Ponte.

Venafro: Centro PMA NASCERE.

Venezia: ARC STER - Centro Studi per la Terapia della Sterilità della Coppia s.r.l.

Verona: Centro Athena; Centro PMA e preservazione della fertilità Ospedale della Donna e del Bambino; Studio Medico "Tethys".

Viareggio: Centro di Riproduzione Assistita "Ettore Barale" - U.O.S. Centro Procreazione Medicalmente Assistita - Ospedale Versilia - Azienda USL Toscana NordOvest.

Vicenza: Centro di Medicina S.p.A.

Villorba: Centro di Medicina S.p.A.

**Kazakhstan**

Actobe: IVF Center " FAMILY DOCTOR AND CO",

Almaty: International Clinical Center for Reproductology “Persona”; LLP “Institute of Reproductive Medicine”; LLP “ECO Center”; LLP “Firma ECOMED”; LLP “Nuray Clinic”

Nur-Sultan: LLP “Astana ECOLIFE”; LLP “Genom Clinic”; KF “UMC”; LLP “ECO CPP”; LLP “Health and Science Center “M1”; LLP “Ecomed plus”

Atyrau: LLP “Ecomed-Atyrau”

Taraz: “Medical Center of Marriage and Family”

Shymkent: “IVF clinic of Dr.Tararaka”; LLP “Ecomed Shymkent”; LLP “Institute of Reproductive Medicine Shymkent”

Karaganda: LLP “Akzhan”

**Latvia**

Riga: Northway klīnika, RMC “Embrions”, EGV Clinic.

**Lithuania**

Vilnius: Vilnius University Hospital Santaros Klinikos Department of Obstetrics and Gynecology Santaros Fertility Center, public clinic; Northway Medical Centers, private clinic; **Grazinos Bogdanskienes Fertility Center, private clinic.**

Kaunas: The Hospital of Lithuanian University of Health Sciences Kauno klinikos Department of Obstetrics and Gynecology Reproductive Medicine Center, public clinic.

Klaipėda: „Jolsana“ Medical Center, private clinic.

**Vilnius/Kaunas/Klaipėda: Vaisingumo Klinika, private clinic.**

**Luxembourg**

Luxembourg: Centre Hospitalier de Luxembour, Centre de Stérilité et de Reproduction

**Malta**

Msida: Mater Dei Hospital ART clinic, public governmental centre for ART

Sliema: St James Hospital, private ART clinic

**Moldova**

Chisinau: Repromed clinic; TerraMed medical center; Medpark hospital

**Montenegro**

Budva: Human Reproduction Centre, Budva, private centre

Cetinje: Human Reproduction Department, Hospital Danilo I Cetinje;

Podgorica: LIFE, private IVF clinic;

Podgorica: ARS MEDICA, private IVF clinic

**North Macedonia**

Bitola: Private Hospital Plodnost

Skopje: Re-Medika; Acibadem Sistina Hospital - Centre for Assisted Reproduction (IVF); Newborn Clinic-Fertility Clinic

Stip: Private Health Centre D-r Organdziski

**Norway**

Bergen: Haukeland Universitetssykehus; Klinikk Hausken

Haugesund: Haugesund Sykehus HF; Klinikk Hausken

Oslo: Livio IVF klinikken Oslo; Oslo Universitetssykehus; Fertilitetssenteret; Klinikk Hausken; Medicus

Porsgrunn: Sykehuset Telemark HF

Stavanger: Klinikk Hausken ; Medicus

Tromsø: Universtitetssykehus Nord Norge

Trondheim: Spiren Fertilitetsklinikk; St Olavs Hospital ; Medicus

**Poland**

Białystok: ARTEMIDA Centrum Ginekologii, Endokrynologii i Medycyny Rozrodu; BOCIAN Klinika Leczenia Niepłodności, Ginekologii i Położnictwa; Klinika Rozrodczości i Endokrynologii Ginekologicznej, Uniwersytecki Szpital Kliniczny; KRIOBANK Centrum Leczenia Niepłodności, Ginekologia i Położnictwo

Bydgoszcz: GENESIS NZOZ Centrum Medyczne

Bytom: ANTRUM Centrum Medyczne, Laboratorium DEMETER Stanisław Horák

Gdańsk: INVICTA Kliniki Leczenia Niepłodności

Gdynia: GAMETA Centrum Zdrowia; INVIMED Europejskie Centrum Macierzyństwa

Katowice: ANGELIUS PROVITA Centrum Medyczne; BOCIAN Klinika Leczenia Niepłodności, Ginekologii i Położnictwa; GYNCENTRUM Klinika Leczenia Niepłodności i Diagnostyki Prenatalnej; INVIMED Europejskie Centrum Macierzyństwa

Kielce: GAMETA Centrum Zdrowia

Kraków: ARTVIMED Centrum Medycyny Rozrodu; GYNCENTRUM Klinika Leczenia Niepłodności i Diagnostyki Prenatalnej; MACIERZYŃSTWO Centrum Medyczne; MEDISTICA Ginekologia i Płodność; PARENS Centrum Leczenia Niepłodności

Łódź: GRAVITA Diagnostyka i Leczenie Niepłodności; SALVE-MEDICA

Lublin: AB OVO NZOZ Centrum Zdrowia Rodziny

Motycz: OVUM Specjalistyczne Centrum Medyczne

Mysłowice: NOVOMEDICA Centrum Leczenia Niepłodności

Niemcz: ZDRÓWKO Klinika

Olsztyn: ARTEMIDA Centrum Ginekologii, Endokrynologii i Medycyny Rozrodu

Opole: PARENS Centrum Leczenia Niepłodności

Płock: GRAVIDA

Poznań: BOCIAN Klinika Leczenia Niepłodności, Ginekologii i Położnictwa; INVIMED Europejskie Centrum Macierzyństwa; Klinika Niepłodności i Endokrynologii Rozrodu Uniwersytet Medyczny im. Karola Marcinkowskiego w Poznaniu; MEDART Ośrodek Diagnostyki i Leczenia Niepłodności

Rzeszów: PARENS

Rzgów: GAMETA Szpital

Szczecin: VITROLIVE Centrum Ginekologii i Leczenia Niepłodności

Warszawa: BOCIAN Klinika Leczenia Niepłodności, Ginekologii i Położnictwa; FERTIMEDICA Centrum Płodności; FERTINA Centrum Medyczne; INVICTA Kliniki Leczenia Niepłodności;

INVIMED Europejskie Centrum Macierzyństwa; nOvum Przychodnia Lekarska; GAMETA Centrum Zdrowia; PROVITA Warszawa; SALVE-MEDICA

Wrocław: INVICTA Kliniki Leczenia Niepłodności; INVIMED Europejskie Centrum Macierzyństwa; POLMEDIS POLAK Klinika Leczenia Niepłodności

**Portugal**

Guimarães: Hospital da Senhora da Oliveira – Guimarães;

Vila Nova de Gaia: Centro Hospitalar de Vila Nova de Gaia / Espinho, EPE - Unidade de Medicina da Reprodução Dra. Ingeborg Chaves;

Vila Real: Centro Hospitalar de Trás-os-Montes e Alto Douro, EPE;

Porto: Centro Hospitalar do Porto, EPE; Centro Hospitalar de São João, EPE; Centro de Genética da Reprodução Prof. Alberto Barros; CETI (Centro de Estudos e Tratamento da Infertilidade); CEIE (Centro de Estudos de Infertilidade e Esterilidade); Procriar (Centro de Obstetrícia e de Medicina da Reprodução);

Espinho: COGE (Clínica Obstétricia e Ginecológica de Espinho);

Braga: Ferticare (Centro de Medicina da Reprodução);

Coimbra: Centro Hospitalar Universitário de Coimbra, EPE; CLINIMER (Clínica de Medicina da Reprodução); FERTICENTRO (Centro de Estudos de Fertilidade);

Covilhã: Centro Hospitalar Cova da Beira, EPE;

Lisbon: Centro Hospitalar Lisboa Norte, EPE - Hospital de Santa Maria; Centro Hospitalar Lisboa Central, EPE - Maternidade Dr. Alfredo da Costa; AVA CLINIC; British Hospital XXI; CEMEARE (Centro Médico de Assistência à Reprodução); Hospital dos Lusíadas; Instituto Extremeño de Reproducción Asistida, Sucursal em Portugal (IERA); IVI Lisboa; GINEMED Lisboa;

Almada: Hospital Garcia de Orta, EPE;

Faro: FERTIMED (Centro Médico de Reprodução Humana);

Portimão: Mediart;

Ponta Delgada: MEKA CENTER (CLÍNICA DA MULHER);

Funchal: Hospital DR. Nélio Mendonça, Sesaram, EPE; FERTIMADEIRA (Centro de Estudos de Fertilidade e de Criopreservação da Madeira).

**Russia**

Abakan: Republican Clinical Perinatal Center

Arkhangelsk: LLC "IVF Center"; LLC “IVF Center on Voskresenskaya”

Astrakhan: LLC "IVF Center"; Region Center of Family Health Care and Reproduction

Barnaul: Siberian institute of human reproduction and genetics; Regional clinical hospital; Clinic "Mother and Child Barnaul"

Belgorod: Regional clinical hospital of St. Joasaph, Department of ART; LLC “Fomin's Clinic”

Blagoveshchensk: LLC "IVF Center"; LLC Clinic “AmurMed”

Bryansk: LLC "IVF Center"; Regional Center of Family Health Care and Reproduction; Bryansk Interdistrict Hospital

Cheboksary: State-financed organization Presidential Perinatal Center of the Ministry of Health and Social Development of the Chuvash Republic; LLC "IVF Center"

Chelabinsk: Municipal Autonomous health care institution "ART Center"; Regional Perinatal Center; Clinic "South Ural State Medical University"; LLC Medical Center "Lotos"; LLC "Family Planning Center"; Center of Family Medicine

Chita: Trans-Baikal regional perinatal center

Ekaterinburg: Ural Research Institute of Maternity and Child Care; Center of Family Medicine; Multifunctional Center "Harmony"; Clinical and diagnostic center "Maternal and child health protection", LLC Clinical Institute of Reproductive Medicine, LLC Clinic "IVF - Partus Center"

Izhevsk: Center for reproductive health; Clinic of Nuriev – Izhevsk; First Republican Clinical Hospital, Center of IVF and Reproduction

Irkutsk: Regional perinatal center, Department of ART; Clinic "Mother and Child Irkutsk"; LLC "IVF Center"

Ivanovo: Clinic of Modern Medicine; Research Institute of Maternity and Childhood n.a. V.N. Gorodkov

Kaliningrad: LLC "Center-Doctor"; LLC "IVF Center"

Kaluga: LLC "IVF Center"; Dr. Fomin's Clinic

Kazan: Clinic "Scandinavia AVA-Kazan"; LLC "Nuriev's clinic"; Kazan branch of LLC "AVA-PETER"; Clinic of Family Medicine

Kemerovo: State Medical Institution "Kuzbass Regional Clinical Hospital n.a. S.V. Belyaev"; LLC "Center for Family Health and Reproduction "Krasnaya Gorka""

Kirov: Regional Clinical Perinatal Center; LLC "Nuriev's clinic - Kirov"

Kislovodsk: Clinic «Elorma»

Khabarovsk: Perinatal Center n.a. Prof. G.S.Postol

Khanty-Mansiysk: District Clinical Hospital

Kostroma: LLC "IVF Center"

Krasnodar: "OXY-Center” Ltd; Clinic of Human Reproduction “Embryo”; Clinic "First word"; Regional Center of Family Health Care and Reproduction; Base Obstetrics and Gynecology Clinic of Kuban State Medical University; LLC "Clinic Ekaterininskaya"; Children's City Clinical Hospital; LLC "Kuban Medical Center"

Krasnoyarsk: Clinic "Mother and Child Krasnoyarsk"; Medical Center Gynecological Endocrinology and Reproduction «Three hearts»

Kurgan: JSC "Family Medicine Center"

Kursk: Regional perinatal center; LLC "IVF Center"

Lipetsk: VITROKLINIK; LLC "IVF Center"

Magnitogorsk: JSC "Family Medicine Center"

Makhachkala: Dagestan Republican Center of Family Health Care and Reproduction

Moscow: Moscow Regional Perinatal Center, ART department; Lapino Clinical Hospital; Medsi Clinic on Solyanka; JSC "European Medical Center", Clinic of Reproduction and prenatal Medicine; City Clinical Hospital n.a. V.V. Veresaev ", ART Department; Moscow Regional Research Institute of Obstetrics and Gynecology; Clinic "Moskvorechye"; Perinatal Medical Center "Mother and Child"; Medical Center for infertility treatment "Embryo"; University Clinic "I am healthy!"; SM-Clinic; GMS ECO; Clinic "Mother and Child Lefortovo"; Clinic "Mother and Child Khodynka field"; Institute of Reproductive Medicine REMEDI; Clinic of assisted reproductive technologies "Test tube baby"; LLC "Clinic of Professor V.M. Zdanovsky"; LLC "Mother and child Southwest"; Clinic "IVF Center"; Center for Reproduction and Genetics “Nova Clinic”; Medical Center “ART-IVF”; Clinic "New life"; NGC Clinic; Clinic MAMA; LLC "Prior Clinic"; Clinic "Biooptima"; Clinic "Mother and Child Kuntsevo"; Reproduction Center "Lifeline", Branch on Kurskaya; Reproduction Center "Lifeline", Branch on Slavyansky Boulevard; Center of Reproduction and Genetics "Fertimed"; Clinic "Altra Vita"; First Moscow State Medical University n.a. I.M. Sechenov; National Medical Research Center of Obstetrics, Gynecology and Perinatology n.a. V.I.Kulakov", Department of Assistive Technologies in Infertility Treatment n.a. B.V.Leonov; National Medical Research Center of Obstetrics, Gynecology and Perinatology n.a. V.I.Kulakov", 1st gynecological department; National Medical Research Center of Endocrinology; Central Clinical Hospital with Polyclinic" of the Office of the President of the Russian Federation; Central Clinical Hospital of the Russian Academy of Sciences; Central Clinical Hospital "RZD-Medicine"

Naberezhnye Chelny: Clinic of Nuriev – Chelny

Nalchik: LLC "IVF Center"

Nizhny Novgorod: Clinic «AIST»; Federal Budget Health Care Institution "Volga district health center"; Clinic “Dad, mom and baby”

Novokuznetsk: Clinic "Media-Service"; Clinic "Mother & Child Novokuznetsk”

Novosibirsk: Medical Center "Avicenna"; Center for reproductive medicine “Mother and Child”; Vitromed Reproductive Health Clinic; Center for New Medical Technologies

Omsk: The fertility clinic "EmBio"; City clinical perinatal center; Omsk Center for Reproductive Medicine; LLC "IVF Center"

Orel: LLC "IVF Center"

Orenburg: Regional Clinical Hospital No. 2; Medical Center for Cell Technologies “New Life”; Clinic “Medgard-Orenburg"; Clinic "MaxiMed"; Classic LLC

Penza: LTd “INMED”

Perm: Reproductive Clinic “Philosophy of Life”; Clinic “Mother and Child Perm”

Petrozavodsk: LLC "IVF Center"

Pskov: LLC "IVF Center"

Rostov-Don: Center of Human Reproduction and IVF; Clinic «Genom-Don»; LLC "IVF Center".

Ryazan: Clinic "Mother and Child Ryazan"; LLC "IVF Center"

Samara: State-Financed Health Facility "Samara Regional Medical Center “Dynasty"; IDK Medical Company - Clinic "Mother and Child Samara"; Clinical Hospital "IDK"; Clinic for reproductive health “IVF”

Saransk: Mordovian Republican Clinical Perinatal Center; "Centre IVF "Gera", LLC

Saratov: Clinical Perinatal center of the Saratov region; Clinic of doctor Paramonov; Professor Churakov's Clinic; Center for Reproductive Technologies "SOVA IVF"

Sevastopol: LLC "IVF Center"

Simferopol: Clinic "Vera"; Genesis Clinic; Company "Ethel"; Medical Clinic "YOUR DOCTOR"; Tavrichesky clinic of reproductive medicine "Ferti-Line"; LLC "IVF Center"

Smolensk: Clinical Hospital No. 1; LLC "IVF Center"

Sochi: The Russian-American center for reproduction and human genetics; Medical Center “Embryo-Sochi”

St.Petersburg: International Center for Reproductive Medicine; Saint-Petersburg State Establishment of health protection «Maternity welfare clinic №44» of Pushkinskiy district; Reproduction and family planning center; Saint-Petersburg State Pediatric Medical University; Baltic Institute of Human Reproductology; “AVA-PETER” Ltd; "Aimed" Ltd; Federal Medical research centre n.a. V. A. Almazov; Euromed-clinic; «Genesis» Ltd; Clinical Hospital # 122 n.a. L.G.Sokolov; Clinic “EmbryLife”; Next Generation Clinic; Clinic "Mother and Child St.Petersburg"; The family planning center "Medica"; Federal state budgetary scientific institution "Research Institute of obstetrics, gynecology and reproduction n.a. D.O.Ott”, Department ART; LLC "International Fertility Center"; LLC “ICLINIC”

Stavropol: Stavropol Regional Clinical Advisory and Diagnostic Center; LLC "IVF Center"

Surgut: Surgut Clinical Perinatal Center

Syktyvkar: Komi Republican Perinatal Center

Tambov: LLC "IVF Center"

Tolyatti: Consultative and diagnostic department Interdistrict perinatal center GBUZ CO "TGKB N 5"; Clinic "Mother and Child Tolyatti"

Tomsk: Genom-Tomsk LLC; Regional Perinatal Center n.a. I.D. Yevtushenko, ART department

Tver: Dr. Fomin's Clinic; Сlinical perinatal center n.a. E.M. Bakunina; Clinic of reproductive medicine and genetics "Genetis"

Tula: Center of new medical technology; VitroClinic; Clinic "Mother and Child Tula"

Tumen: International Medical Center for Reproduction "Mercury"; Medical Center "Malysh"; Clinic "Mother and Child Tumen"

Ulan-Ude: DiaGroup Medical Center LLC

Ulyanovsk: Аlliance Сlinic LLC; LLC "IVF Center"

Ufa: Republican Medical and Genetic Center; Medical Center “Family”; Clinic "Health of women and men"; Clinical hospital "Mother and Child. Ufa"

Vladikavkaz: Republic Center of Family Health Care and Reproduction

Vladivostok: LLC Women's Health Clinic "Santa Maria"

Veliky Novgorod: LLC “IVF Center”

Vladimir: LLC "IVF Center"

Volgograd: Volgograd State Medical University, Clinic №1; Clinic “Genom-Volga”, Clinic "Mother and Child Volgograd”, LLC "IVF Center"

Vologda: LLC "IVF Center"

Voronezh: Voronezh Region Clinical Hospital №1, LLC "IVF Center"

Yakutsk: Republic Hospital N1 – National Centre of Medicine, Department of ART

Yaroslavl: Regional Perinatal Center; Clinic "Mother and Child Yaroslavl "

**Serbia**

Belgrade: Clinical Center of Serbia; Clinic "Narodni front"; Clinical Center Kragujevac;

Novi Sad: IVF Center GINS; SGH Genesis

Valjevo: IVF Center BB

**Slovakia**

Bratislava: University Center for Reproductive Medicine, Ferticent, Iscare, GynFiv, Repromedica, Reprofit, Helios

Nitra: Gyncare

Žilina: GynFiv

Martin: Helios, Iscare

Banská Bystrica: Helios

Košice: Gyncare, SPLN, Helios

**Slovenia**

Ljubljana: Department of Human Reproduction, Division of Gynaecology, University Medical Centre Ljubljana,

Maribor: Department of Reproductive Medicine and Gynaecologic Endocrinology, University Medical Centre Maribor,

Postojna: Centre for Infertility Treatment Postojna

**Spain**

A Coruña: Equipo Ron – Hospital Quirón A Coruña; HM Belén; Clínica Segrelles

Albacete: Consultorio de Ginecología y Obstetricia; H. General De Albacete; Instituto Bernabéu ALBACETE

Alcalá De Henares: Dr. Goya Analisis S.L.; H. Principe De Asturias; Unimequi

Alcázar de San Juan: Ginequalitas SLP

Alcorcón: ReproFiv

Algeciras: Clínica Medrano

Alicante: Ferrobelab, S.L.; H. General De Alicante; Instituto Bernabéu; IVF-SPAIN (Alicante); IVI Alicante; Unidad de Reproducción Clínica Vistahermosa; Accuna.

Almería: C.H. Torrecárdenas; IVI Almeria; Unidad de Reproducción - Hospital Mediterráneo

Almoradí: Clínica Ufeal

Aravaca: URH Garcia Del Real

Badajoz: CERHA; Instituto Extremeño de Reproducción Asistida

Baracaldo: Hospital Universitario Cruces

Barcelona: C.I.R.H; Centro de reproducción asistida clínica Sagrada Familia; Centro Medico Teknon; Clínica EUGIN; Fertilab.Institut Catalá de Fertilitat; Fundació Puigvert - Hospital de la Santa Creu i Sant Pau; General Lab; Gine-3; Gravida; H. Del Mar; Hospital Clínic de Barcelona; Hospital Quiron Barcelona; Hospital Valle Hebrón; Institut Dexeus; Institut Marques; IVI Barcelona; Girexx Barcelona - Fertility Clínics SL.; Fertty; FECUNMED-Hospital Universitari Sagrat Cor

Benalmádena: IVI Malaga

Beniarbeig: IREMA

Benidorm: IMED Hospitales

Bilbao: Clínica Ginecológica Bilbao; Consultorio Ginecologico Elcano; Instituto IGIN; Quiron Bilbao; Reproducción Bilbao; Clínica Euskalduna

Boadilla del Monte: HM Fertility Center Montepríncipe

Burjassot: UR IMED Valencia.

Cáceres: Clínica Norba

Cádiz: Clínica La Salud; H. U. Puerta del Mar

Carcaixent: FIVIR

Cartagena: Instituto Bernabéu Cartagena, SL.; IVI Cartagena

Castellón: H. General De Castelllon; Hospital Rey Don Jaime; IVI Castellón

Ciudad Real: Clínica Rubal; QuirónSalud Ciudad Real

Ciutadella de Menorca: IBILAB Menorca

Córdoba: Clínica Bau - Cordoba; Clínica IFEM; Clínica Povedano; H. U. Reina Sofia

El Ejido: Clínica JOFRE-FIV

El Palmar: Hospital Clínico Universitario Virgen de la Arrixaca

Elche: H. General De Elche; In Vitam Centro de Medicina Reproductiva

Figueres: FIV Obradors

Fuengirola: Clínica Fertia

Getafe: H. De Getafe; Instituto Para El Estudio De La Esterilidad

Gijón: FIV4-Instituto de Reproducción Humana Gijón; Clínica Ergo Biotech SL

Girona: Centro De Genética Girona; Girexx (FIV Girona SLP); Hospital Universitari De Girona Doctor Josep Trueta; Unitat de Reproduccio Humana i Diagnostìc Genètic. Clínica Girona

Guadalajara: FIV Laber

Granada: Clínica Sanabria; H.U. Virgen de las Nieves; Instituto Avantia de Fertilidad; MAR&Gen; UR HOSPITAL INMACULADA; Vithas Hospital la Salud; Aluz, Clínica de Fertilidad Avanzada; Clínica Pedrosa; Clínica INAGOR

Granollers: FECUNMED

Huelva: Hospital Costa de la Luz; Hospital Vázquez Díaz

Ibiza: Hospital Can Misses; IBILAB Pititusses

Jaén: CARHA; Ciudad de Jaén

Jerez De La Frontera: ULTRAFIV-BAHIA S.L.; Clínica Serman; Clínica Beiman

La Cañada: Unidad de Reproducción Hospital Virgen del Mar

La Laguna: Centro de Asistencia a la Reproducción Humana de Canarias; H. Universitario De Canarias

Las Palmas de Gran Canaria: CIRA Las Palmas; H.U.Materno Infantil de Las Palmas de Gran Canaria; Instituto Canario De Infertilidad, S.L.; IVI Las Palmas; CIRA Las Palmas

Leioa: IVI Bilbao

León: Centro Asistencial Universitario de León( CAULE ); Centro Ginecológico de León

Lleida: Avantmedic Unitat de la Dona; FIv Lleida; CEFER Lleida

Logroño: Centro Ginecologico Manzanera; Clínica Alxen . Centro Ginecológico Riojano; Clínica Ginecológica Juana Hernández; Ginesalud; H. San Pedro

Lugo: EVALO Centro Ginecológico

Madrid: Centro Médico Milenium Alcobendas Sanitas; Clínica Dr. Eduardo Cubillo; Clínica Ruber Internacional; Clínica Ruber-Centro de Reproducción Madrid, S.L.; Clínica Tambre; EVA QX Lab, S.L; FivMadrid; Fundacion Jimenez Diaz UTE; GINEFIV; H. 12 De Octubre; H. Ramon y Cajal; Hospital Clínico San Carlos; Hospital General Universitario Gregorio Marañón; Hospital La Paz; Hospital Nuestra Señora de América; Hospital Universitario Moncloa S.A.U; Instituto Europeo de Fertilidad; Instituto Ginecologico 'La Cigüeña'; Instituto Madrileño de Fertilidad; IVI Madrid; MINIFIV; Procreatec; Clínica EasyFIV; Eugin; Amnios In Vitro Projec

Málaga: Centro Gutenberg; H. Materno Infantil De Malaga - Carlos Haya; Hospital Quiron Málaga; IMAFER – Clínica Victoria; Instituto Malavé de Reproducción; Malaga F.I.V.; UR El Ángel

Marbella: CERAM (Centro De Reproducción Asistida De Marbella); FIV Marbella; HC-Fertility; Hospital Costa de Sol; Hospital Ochoa

Mataro: IMARA

Melilla: Clínica Ginecológica Dr. Marín; Clínica Imera

Móstoles: HM Fertility Center Puerta del Sur

Murcia: Centro Ginecológico de Fertilidad y Genética; Fertilidad Roca; Imar Fertilidad; Instituto de Reproducción Asistida QuironSalud Dexeus Murcia; IVI Murcia; Tahe Fertilidad; Unidad de Reproducción La Vega

Oviedo: CEFIVA - Oviedo; FIV4-Instituto de Reproducción Humana; H. UCA, Unidad reproducción, Hospital materno-infantil

Palamós: Serveis de Salut Integrats Baix Empordà

Palma De Mallorca: CEFIVBA: Centro Fecundacion In Vitro Balear; FIV de Mallorca; Hospital Universitario Son Espases; H. Fundación Son Llatzer; Instituto de Fertilidad; IVI Illes Balears; Instituto Bernabeu

Pamplona: Complejo Hospitalario de Navarra; Estudio Médico Navarro; Quirón Pamplona

Pozuelo de Alarcón: Hospital Universitario Quirónsalud Madrid

Reus: Biogest; Conceptum; Procrear

Rincón De La Victoria: Instituto de Fertilidad Clínica Rincón

Roquetas De Mar: Roquetas FIV

Salamanca: Clínica Mencía

Salt: Hospital de Santa Caterina. Salt

San Sebastián: Clínica Zuatzu; Hospital Quirón Donostia; Hospital Universitario de Donostia; Instituto Vasco de Fertilidad Donostia; IVI San Sebastian / Donostia

Sant cugat del Valles: Áptima Centre Clinic Mutua de Terrasa

Santa Cruz de Bezana: FIVSantander

Santa Cruz De Tenerife: Centro De Endocrinologia De La Reproducción de Tenerife; Centro Madre (Centro Mahatni de Reproducción); H. Nuestra Sra. De La Candelaria; Irmo, S.L.

Santander: CER Santander; IVI Santander; URA Valdecilla. Hospital U .Marques de Valdecilla

Santiago de Compostela: Complexo Hospitalario Universitario de Santiago; Unidad de Reproducción Asistida La Rosaleda; Zygos, Centro Gallego de Reproducción

Sevilla: Caremujer- Quiron Sagrado Corazón; Embryocenter; Ginemed; H. Universitario Virgen De Valme; Hospital Victoria Eugenia (INEBIR); Hospital Virgen del Rocío; IVI Sevilla; MasVida Reproducción; Hospital Quirón Infanta Luisa

Tarragona: Embriogyn

Toledo: H. Virgen De La Salud; HM IMI Toledo

Torremolinos: URA Clínica Santa Elena

Valencia: Clínica Quiron Valencia; CREA Valencia; Equipo Juana Crespo; FIV Valencia; H. Arnau De Vilanova; H. Dr. Peset Aleixandre; H.U. La Fe; Hospital Clínico Universitario de Valencia; Hospital General Universitario de Valencia; Imer; IVI Valencia S.L; Policlínico Valencia; UHRA Nisa-Ginemed

Valladolid: Fiv Madrid Valladolid; FIV Recoletos Valladolid; Hospital Universitario Rio Ortega; Unidad Reproducción, Servicio Ginecología y Obstetricia del H.C.U. De Valladolid

Vic: Unitat Endocrinologia Ginecològica

Vigo: Centro Hospitalario Universitario de Vigo; Centro Medico Pintado; Hospital Nuestra Señora de Fátima; IVI Vigo; Clínica Nida

Vitoria: ART Vitoria; Hospital Universitario Araba sede Txagorritxu

Zaragoza: Centro Médico Ginfer SLP; Clínica Ginecológica de Zaragoza S.L.; Clínica Gobest; Clínica Montpellier (UR Montpellier); H. Clínico Zaragoza; H. Miguel Servet; IVI Zaragoza; Quirónsalud Zaragoza

**Sweden**

Falun: Livio Falun

Göteborg: Livio Göteborg; Reproduktive medicin Sahlgrenska University Hospital; Nordic IVF Göteborg

Linköping: RMC Linköping University Hospital.

Malmö: Livio Malmö; RMC Malmö University Hospital; Nordic IVF Malmö

Örebro: IVF unit Örebro University Hospital

Stockholm: Livio Kungsholmen; IVF unit Karolinska University Hospital, Huddinge; IVF unit Sophiahemmet; Livio Gärdet; Nordic IVF Stockholm, Stockholm IVF

Umeå: Livio Umeå

Uppsala: Carl von Linné clinic; Reproductive center, Academiska University Hospital; Cmedical Fertilitet Gynhälsan

**Switzerland**

Baden: Kinderwunschzentrum Baden "Täfernhof“

Basel: Universitäts - Frauenklinik Basel; Kinderwunschzentrum Regio Basel; Fertilitas

Bellinzona: Endomed

Bern: Kinderwunschzentrum Inselspital Bern; IVF-Team Lindenhofspital;

Biel : Care - Centrum für assistierte Reproduktionsmedizin und gynäkologische Endokrinologie

Chur: Kinderwunschzentrum Fontana

Freiburg : Centre de Procréation médiclement assistée

Genève: : Ferti Genève (Centre PMA Clinique Générale Beaulieu-UNILABS; Hôpitaux Universitaire de Genève); Centre Clinique des Grangettes ; BabyImpulse (Centre Clinique des Grangettes ; Medixy)

Kreuzlingen: Milagro

Lausanne: Centre de procréation médicalement assistée (CPMA);  Unité de Médecine de la Reproduction et d’Endocrinologie gynécologique - CHUV

Locarno: Centro Cantonale di Fertilità

Lugano: ProCrea

Luzern: Kinderwunschzentrum Kantonsspital Luzern; Kinderwunschzentrum Klinik St. Anna Hirslanden

Olten: Fertisuisse

St Gallen: FIORE- Fachinstitut für Reproduktionsmedizin und gynäkologische Endokrinologie

Schwyz: Kopelli Klinik

Zürich: GYN-A.R.T. AG; Kinderwunschzentrum Universitätsspital Zürich; Gyné Invitro; OVA - IVF Clinic Zürich; 360 Grad Kinderwunsch Zentrum; Admira Kinderwunschzentrum

**The Netherlands**

Amsterdam: Academisch Medisch Centrum; Vrije universiteit Medisch Centrum

Elsendorp: Nij Geertgen

Groningen: Universitair Medisch Centrum Groningen

Hengelo: Fertiliteitskliniek Twente

Leiden: Leids Universitair Medisch Centrum

Leiderdorp: Medisch Centum Kinderwens

Maastricht: Universitair Medisch Centrum Maastricht

Nijmegen: Universitair Medisch Centrum Nijmegen

Rotterdam: Erasmus Medical centre

Tilburg: Elisabeth Twee Steden Ziekenhuis

Utrecht: Universitair Medisch Centrum Utrecht

Voorburg: Reiner de Graaf Groep

Wolvega: Nij Barrahûs

Zwolle: Isala

**Turkey**

Adana: Baskent University, Adana, Dr. Turgut Noyan Application and Research Center,

Ankara: University of Health Sciences Ankara Etlik Zubeyde Hanim Obstetrics and Gynecology Health Application and Research Center, Hacettepe University Faculty of Medicine IVF Unit, Ankara University Faculty of Medicine Reproductive Health Diagnosis Treatment and Application Center, Anatolia IVF and Women's Health Center, Private Ankara IVF Center, Gazi University Faculty of Medicine IVF Unit, Gen-Art IVF Women's Health and Reproductive Biotechnology Center

Antalya: Akdeniz University Faculty of Medicine IVF Unit, Gelecek IVF Center,

Bursa: Uludag University Faculty of Medicine IVF Unit, Eurofertil IVF Center,

Denizli: Private Health Hospital,

Istanbul: Bahceci Health Group, Acibadem Health Group, Vehbi Koc Foundation Health Institutions (Koc University Reproductive Medicine and IVF Center, American Hospital Reproductive Medicine and IVF Center), Istanbul University Faculty of Medicine IVF Unit,

Izmir: Tepecik Training and Research Hospital IVF Unit

**Ukraine**

Chernivtsi: Medical center of infertility treatment

Dnipro: “Intersono” LTD; «Medical Plaza»; LTD «Aurora IVF»

Ivano-Frankivsk: Precarpacian Centre of Reproductive Medicine; Clinic of Reproductive Medicine Extramed; Medical Reproductive Health Centre Damia

Kharkiv: Academician V.I.Grishchenko Clinic for Reproductive Medicine; “ART-clinic”; LTD “Sana-med”; State institution “Ukrainian medical center of obstetrics, gynecology and reproductology of Ministry of public health of Ukraine”

Khmelnytskyi: Olena Vavrynchuk Reproductive Clinic

Kyiv: “Mother and Child” clinic; A.A. Partners; “Rodynne dzherelo” Clinic; NADIYA Clinic of Reproductive Medicine; Isida IVF clinic; “Victoria” Reproductive Genetics Clinic; Clinic of reproductive technologies USIR SNMAPE; LTD “Institute of Reproductive Medicine”; Institute of Genetic Reproduction; “Biotexcom” LTD; Medical Cneter “Nativita”; Institute for Family Planning; Center for Reproductive Medicine “MATERI CLINIC”; LTD "Mini-EKZ Center"; LTD “Academiс medical center”; Medical Center “IVF Laboratory”, LTD “Sana-Med Kiev”; Kyiv City Center for Reproductive and Perinatal Medicine; MC "Institute of General Practice - Family Medicine"

Lutsk: Center for Reproductive Medicine BOGOLYUBY

Lviv: IVF clinic “Alternatyva”; Center for Infertility Treatment «Parens-Ukraine»; IVF clinic “Intersono”, Medical Center “Clinic of prof. S. Khmil”(Lviv), “Mother and Child” clinic  (Lviv)

Odesa: REMEDI Center for Reproductive Medicine; “Gameta” Reproductive Health clinic; LADA Reproductive Health Clinic; Reproductive medicine department, University clinic, Odessa national medical university; Medical Center “Academium”, LTD MC “NADIYA Odessa”

Rivne: LTD Medical Center “Blagodar”; "Regional Clinical Medical and Diagnostic Center named after V. Polishchuk"

Ternopil: Medical Center “Clinic of prof. S. Khmil”

Vinnitsa: Medical Center “Vinukrmed”

Zaporizhzhia: Regional Center for rehabilitation of reproductive health

Zhitomir: LTD “MC Materna”

**United Kingdom**

Aberdeen: Aberdeen Fertility Centre

Airdrie: Lanarkshire Acute Hospital NHS Trust

Bath: CARE Fertility Bath

Belfast: Regional Fertility Centre, Belfast; TFP Belfast Fertility

Birmingham: Birmingham Women's Hospital; CREATE Fertility, Birmingham; The Priory Hospital; CREATE Fertility Birmingham

Borehamwood: IVF London

Brighton & Hove: Agora Clinic Brighton; Brighton Fertility Associates

Bristol: Bristol Centre for Reproductive Medicine; CREATE Fertility Bristol; Reproductive Medicine Clinic, Bristol

Bromley: Kent Fertility Center

Cambridge: Bourn Hall Clinic (Colchester); Cambridge IVF

Cardiff: London Women’s Clinic, Wales; Wales Fertility Institute – Cardiff

Carshalton: Beginnings at Epsom & St Helier NHS University Trust

Cheadle: Manchester Fertility

Cheshire: Hewitt Fertility Centre, Knutsford

Cheshunt: Herts and Essex Fertility Centre

Chester: CARE Fertility Chester

Coventry: Centre for Reproductive Medicine, Coventry

Croydon: Fertility in Community

Daresbury: IVF-Life United Kingdom

Darlington: London Women’s Clinic, Darlington

Derby: Royal Derby Hospital

Dundee: Ninewells Hospital

Eastbourne: Sussex Downs Fertility Centre

East Yorkshire: Hull and Est Riding Fertility

Edinburgh: Edinburgh Fertility Centre

Epsom: NewLife Fertility Centre

Essex: TFP Simply Fertility

Exeter: Fertility Exeter

Gateshead: The Gateshead Fertility Unit

Glasgow: Glasgow Royal Infirmary; TFP GCRM Fertility

Kilmarnock: Ayrshire Fertility Unit, Crosshouse Hospital

Kingston: Kingston Hospital ACU

Leeds: Care Fertility Leeds

Leicester: Leicester Fertility Centre; X&Y Fertility

Liverpool: Hewitt Fertility Centre

Llantrisant: Centre for Reproduction & Gynaecology Wales (CRGW)

London: Andrology Solutions; Assisted Reproduction and Gynaecology Centre; Barts Health Centre for Reproductive Medicine; TFP Boston Place; CARE Fertility London; Chelsea & Westminster Hospital; City Fertility; CREATE Fertility, London St Paul's; Guys Hospital; Harley Street Fertility Clinic; Homerton Fertility Centre; IVI London (Wimpole Street); King’s Fertility; London Fertility Centre; London Women's Clinic; Reproductive Genetics Institute; The Centre for Reproductive and Genetic Health; The Evewell Harley Street; The Fertility & Gynaecology Academy; The Fertility Centre at Whittington Health; The Lister Fertility Clinic; Wolfson Fertility Centre - Hammersmith Hospital; Bourn Hall Clinic; London Sperm Bank (LSB) London Bridge; University College London Hospitals

Maidenhead: TFP Thames Valley Fertility

Manchester: CARE Fertility Manchester; Manchester Fertility; St Mary's Hospital

Middlesbrough: The James Cook University Hospital

Newcastle Upon Tyne: Newcastle Fertility Centre at Life

Northampton: CARE Fertility Northampton

Norwich: Bourn Hall Clinic Norwich

Nottingham: CARE Fertility Nottingham; NUH Life Fertility Services; TFP NURTURE Fertility

Oxford: TFP Oxford Fertility

Plymouth: Centre for reproduction and embryology Wales and West

Port Talbot: Wales Fertility Institute-Neath

Putney: Concept Fertility

Romford: Fertility unit Barking, Havering and Redbridge Hospitals trust

Salisbury: Salisbury Fertility Centre

Sheffield: CARE Fertility Sheffield; Jessop Fertility

Shrewsbury: Shropshire and Mid-Wales Fertility Centre

Southampton: Complete Fertility Centre Southampton; TFP Wessex Fertility

Sunderland: Sunderland Fertility Center

Tamworth: CARE Fertility Tamworth

Truro: Cornwall centre for reproductive Medicine (CCRM)

Tunbridge Wells: CARE Fertility Tunbridge Wells

Wickford: Bourn Hall Clinic Wickford

Wigan: Fertility Fusion

Wilmslow: CREATE Fertility, Manchester;

Wimbledon: CREATE Fertility, London Wimbledon;

Woking: CARE Fertility Woking

Wolverhampton: St Jude's Women’s Hospital
